# Establishment and Characterization of a Reliable Xenograft Model of Hodgkin Lymphoma Suitable for the Study of Tumor Origin and the Design of New Therapies

**DOI:** 10.3390/cancers10110414

**Published:** 2018-10-31

**Authors:** Radhia M’kacher, Monika Frenzel, Mustafa Al Jawhari, Steffen Junker, Corina Cuceu, Luc Morat, Anne-Laure Bauchet, Lev Stimmer, Aude Lenain, Nathalie Dechamps, William M. Hempel, Geraldine Pottier, Leonhard Heidingsfelder, Eric Laplagne, Claire Borie, Noufissa Oudrhiri, Dima Jouni, Annelise Bennaceur-Griscelli, Bruno Colicchio, Alain Dieterlen, Theodore Girinsky, Raphael Boisgard, Jean Bourhis, Jacques Bosq, Thomas Mehrling, Eric Jeandidier, Patrice Carde

**Affiliations:** 1 Radiobiology and Oncology Laboratory, CEA, iRCM, University Paris-Saclay, 92 265 Fontenay aux Roses, France; monika.frenzel@hotmail.com (M.F.); mustafa.aljawhari@hotmail.fr (M.A.J.); cuceu_corina@yahoo.com (C.C.); luc.morat@cea.fr (L.M.); audelenain@yahoo.fr (A.L.); williamhempel824@gmail.com (W.M.H.); 2 Cell Environment, Oncology Section, 75020 Paris, France; 3 Institute of Biomedicine, University of Aarhus, DK-8000 Aarhus C, Denmark; sjunker@biomed.au.dk; 4 Platform for Experimental Pathology PathEX/CRC MIRCen/CEA-INSERM, University Paris-Saclay, 92265 Fontenay aux Rroses, France; anne-laure.bauchet@sanofi.com (A.-L.B.); lev.stimmer@cea.fr (L.S.); 5 Platform for Cell Sorting, CEA, iRCM, 92265 Fontenay aux Roses, France; nathalie.dechamps@cea.fr; 6 Laboratoire d’Imagerie Moléculaire Expérimentale Groupe d’Imagerie du Petit Animal CEA/DSV/I2BM/SHFJ/U1023, University Paris-Saclay, 91400 Orsay, France; geraldine.pottier@cea.fr (G.P.); raphael.boisgard@cea.fr (R.B.); 7 MetaSystems GmbH, Robert-Bosch-Str. 6D, 68804 Altlussheim, Germany; lheidingsfelder@metasystems.de; 8 Pole Concept, 75016 Paris, France; eric.laplagne@gmail.com; 9 APHP-Hopital Paul Brousse Université Paris Sud/ESteam Paris Inserm UMR 935, 94800 Villejuif, France; claire.borie@aphp.fr (C.B.); noufissa.oudrhiri@aphp.fr (N.O.); dimajouni@yahoo.fr (D.J.); annelise.bennaceur@pbr.aphp.fr (A.B.-G.); 10 IRIMAS, Institut de Recherche en Informatique, Mathématiques, Automatique et Signal, Université de Haute-Alsace, 68093 Mulhouse, France; bruno.colicchio@uha.fr (B.C.); alain.dieterlen@uha.fr (A.D.); 11 Department of Radiation Oncology, Gustave Roussy Cancer Campus, University Paris-Saclay, 94805 Villejuif, France; theogirinsky@me.com (T.G.); Jean.Bourhis@unil.ch (J.B.); 12 Departement of Anapathology, Gustave Roussy Cancer Campus, University Paris-Saclay, 94805 Vilejuif, France; jacques.bosq@gustaveroussy.fr; 13 Mundipharma-EDO GmbH, CH-4020 Basel, Switzerland; thomas.mehrling@mundipharma-edo.com; 14 Department of Genetic, Groupe Hospitalier de la Région de Mulhouse Sud-Alsace, 68093 Mulhouse, France; jeandidiere@ghrmsa.fr; 15 Department of Medicine, Gustave Roussy Cancer Campus, University Paris-Saclay, 94805 Villejuif, France; dr.pcarde@gmail.com

**Keywords:** Hodgkin lymphoma, animal model, CD30−/CD15−, telomere dysfunction, telomerase, EDO-S101

## Abstract

To identify the cells responsible for the initiation and maintenance of Hodgkin lymphoma (HL) cells, we have characterized a subpopulation of HL cells grown in vitro and in vivo with the aim of establishing a reliable and robust animal model for HL. To validate our model, we challenged the tumor cells in vivo by injecting the alkylating histone-deacetylase inhibitor, EDO-S101, a salvage regimen for HL patients, into xenografted mice. *Methodology:* Blood lymphocytes from 50 HL patients and seven HL cell lines were used. Immunohistochemistry, flow cytometry, and cytogenetics analyses were performed. The in vitro and in vivo effects of EDO-S101 were assessed. *Results:* We have successfully determined conditions for in vitro amplification and characterization of the HL L428-c subline, containing a higher proportion of CD30−/CD15− cells than the parental L428 cell line. This subline displayed excellent clonogenic potential and reliable reproducibility upon xenografting into immunodeficient NOD-SCID-gamma (−/−)(NSG) mice. Using cell sorting, we demonstrate that CD30−/CD15− subpopulations can gain the phenotype of the L428-c cell line in vitro. Moreover, the human cells recovered from the seventh week after injection of L428-c cells into NSG mice were small cells characterized by a high frequency of CD30−/CD15− cells. Cytogenetic analysis demonstrated that they were diploid and showed high telomere instability and telomerase activity. Accordingly, chromosomal instability emerged, as shown by the formation of dicentric chromosomes, ring chromosomes, and breakage/fusion/bridge cycles. Similarly, high telomerase activity and telomere instability were detected in circulating lymphocytes from HL patients. The beneficial effect of the histone-deacetylase inhibitor EDO-S101 as an anti-tumor drug validated our animal model. *Conclusion:* Our HL animal model requires only 10^3^ cells and is characterized by a high survival/toxicity ratio and high reproducibility. Moreover, the cells that engraft in mice are characterized by a high frequency of small CD30−/CD15− cells exhibiting high telomerase activity and telomere dysfunction.

## 1. Introduction

Hodgkin lymphoma (HL) is a malignancy of the immune system, characterized by the presence of scarce malignant cells, termed Hodgkin and Reed–Sternberg cells (HRS), which in most cases are derived from germinal center B cells [1]. The poor in vitro growth of malignant lymphocytes from HL lymph nodes and the lack of reliable HL animal models present a major obstacle for studies on molecular mechanisms of HL development and the identification of putative therapeutic targets. Consequently, such studies rely heavily on cell lines derived from malignant HRS cells [2].

Although all established HL cell lines may give rise to solid tumors at the site of injection following subcutaneous or intraperitoneal injection into NOD/SCID mice [3,4,5,6,7], few reproducible and robust animal models of HL have been described. Xenografted HL cell lines, as well as primary human tumor cells, are only poorly able to generate mediastinal tumors and promote tumor dissemination (50% and 23%, respectively) [4,8]. Indeed, only one previous model system, based on the L540 and L540cy T-cell-derived HL lines, has been successfully applied to the standardization of new molecules for the treatment of HL following the induction of mediastinal tumors and tumor dissemination [9,10,11].

More than 30 years ago, Newcom et al. [12] reported that the L428 HL cell line contains a minor population of clonogenic B cells which appear to be responsible for the generation of HRS cells and the continuous growth of the cell line. Later, B-cell subpopulations (<1%) responsible for generating and sustaining the predominant HRS cell population were identified in HL cell lines (L428, KMH2), HL lymph nodes, and the peripheral blood of newly diagnosed HL patients [13]. It was proposed that small, morphologically normal, CD30− B-cells with chromosomal aberrations are the progenitor cells of the malignant cell fraction in HL [14,15]. However, the low frequency of these cells in HL patients and the central importance of a specific micro-environment required for the growth of HL cells [16], have precluded the generation of an HL animal model based on direct injection of these cells [13]. In addition, the clonogenicity of these cells has been challenged [17,18], weakening the conclusion that these cells might play a major role in the initiation of the disease and subsequent therapy. Recently, circulating HL-specific immunoglobulin gene segments were detected in the peripheral blood at initial diagnosis and during follow-up [19]. It has been well documented that mononuclear Hodgkin cells have a higher proliferation potential than RS cells [20]. Our more recent understanding of the possible role of CD30−/CD15− B cells as precursors of Hodgkin and HRS cells may also provide a more solid basis for establishing an animal model and defining rational therapeutic strategies.

Here, we used the L428 cell line to establish a xenograft HL animal model. We defined conditions for the establishment of an animal model using in vitro expanded L428 clones. They were selected after in vitro expansion of L428 cells and transplanted into immunodeficient NSG mice. The injected clones were highly enriched in CD30−/CD15− cells. Our HL animal model is highly reproducible, without side effects, and the mice show long survival. Seven weeks after injection, immunophenotype analysis showed that the first cells to engraft were characterized by a high frequency of CD30−/CD15− small cells. After in vitro culture, as well as in vivo establishment in the mice, the malignant cells displayed a diploid character, along with a high frequency of telomere loss and telomerase expression. The latter led to dicentric chromosome formation and breakage/fusion/bridge cycles, thus contributing to chromosomal instability in these cells. Interestingly, telomerase activity and telomere dysfunction were also detected in peripheral blood lymphocytes of some HL patients, underscoring the relevance of our findings and of our animal model. Moreover, the successful effect of EDO-S101 on xenografted HL cells in mice validates its potential use for the assessment of novel treatments for HL using an animal model based on transplantation of L428 HL subline cells into NSG mice. Finally, we provide the first proof of concept for the transformation of CD30−/CD15− B cells to HRS cells, suggesting that these cells may represent HL “cancer stem cells” and thus be the true therapeutic target in HL.

## 2. Results

### 2.1. CD30−/CD15− HL Cells Can Acquire a Phenotype Similar to That of Their Parental HL Cell Lines

The role of CD30−/CD15− cells present in HL cell lines has been the subject of debate for several years [12,13,20]. CD30−/CD15− B cells represented up to 0.3% of the cells in the KMH2 and L428 cell lines by flow cytometry and immunofluorescence microscopy (Supplementary Figure S1) [13]. Hampered by the difficulty of isolating and characterizing these very small subpopulations in the HL cell lines, we first tested the colony formation capacity in semi-solid medium (methylcellulose) of seven HL cell lines. Colonies were scored after two weeks, collected and serially re-plated (Supplementary Table S1). We successfully isolated and expanded clones from the L428, KMH2, and L540 cell lines. The sublines derived from these clones had very high cloning efficiency, high frequency of CD30−/CD15− cells as well as a high proliferation capacity relative to their parental cell lines (Supplementary Figure S2). In our hands, other HL cell lines (L1236, HDLM2, L591 and SUP-HD) did not proliferate in methylcellulose.

**Figure 1:**
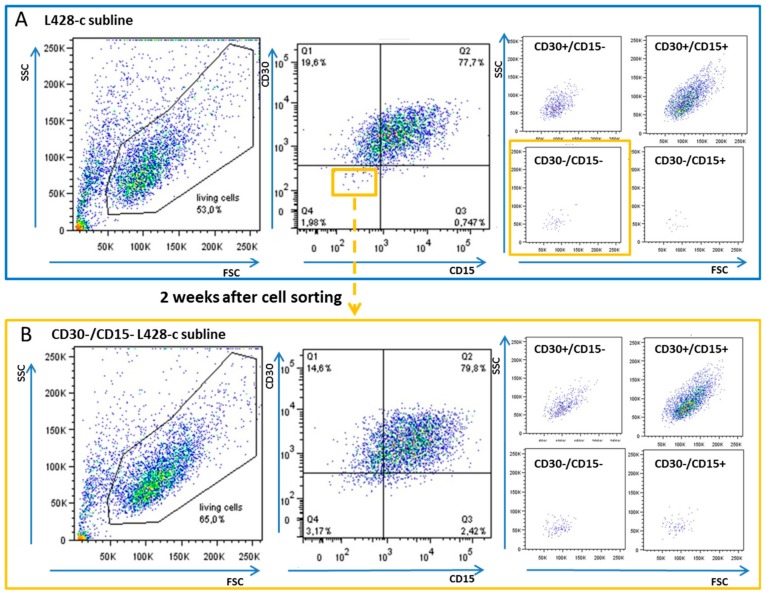
CD30−/CD15− cells from the L428-c subline acquire a phenotype similar to that of the parental cell lines. The proportion of cell-surface forward-scattered (FSC) vs. side-scattered (SSC) signal is presented for the L428-c subline and each subpopulation according to CD30 and CD15 staining. (**A**) L428-c subline cells were stained for CD15 and CD30 and CD30−/CD15− cells (yellow insert) were sorted by fluorescence-activated cell sorting. (**B**) CD30−/CD15− cells from the L428-c subline after two weeks of in vitro culture, showing a similar profile to that of the L428-c subline. There was a higher proportion of CD30−/CD15− cells in the negative sorted cells after two weeks of in vitro culture than in the L428-c subline (3.17% vs. 1.98%). The CD30−/CD15− cells in the L428-c subline, as well as those in the sorted cell population sorting after in vitro culture, were small. Three independent cell-sorting experiments were performed.

Next, we assessed the ability of CD30−/CD15− cells derived from clones to acquire a phenotype similar to that of the parental cell lines. CD30−/CD15− cells were collected by fluorescence-activated cell sorting and analyzed at various time intervals during in vitro culture. After two weeks of culture, CD30−/CD15− cells from L428 acquired a phenotype similar to that of L428-subline, but with a greater clonogenic expansion and higher proliferative index. One of these growing clones termed L428-c was selected and expanded for further experiments (Figure 1). The same approach was applied to clones of the L540 cell line, but their growth was much slower than that of the cells derived from L428-c (Supplementary Figure S3).

### 2.2. Ability of L428-c Subline to Engraft in NSG Mice

Initially, we injected 10^6^ cells of L428, L428-c, KMH2-c, L540-c, L591, or L1236 per NSG mouse to assess the ability of the different HL cell lines to engraft in NSG mice. Of note, 100% (12/12) of the mice injected with the L428-c subline displayed tumor infiltration, whereas this was true for only 77% (7/9) of those injected with the L428 parental cell line, 66% (6/9) of those injected with KMH2-c cells and 66% (6/9) of those injected with L540-c. There was no tumor infiltration in mice injected with L591, and it occurred only at the site of injection in mice injected with L1236 (Supplementary Table S2).

Based on the proliferation rate, the clonogenicity index, and the efficiency to engraft in NSG mice, we chose the L428-c subline to establish the xenograft model.

**Figure 2:**
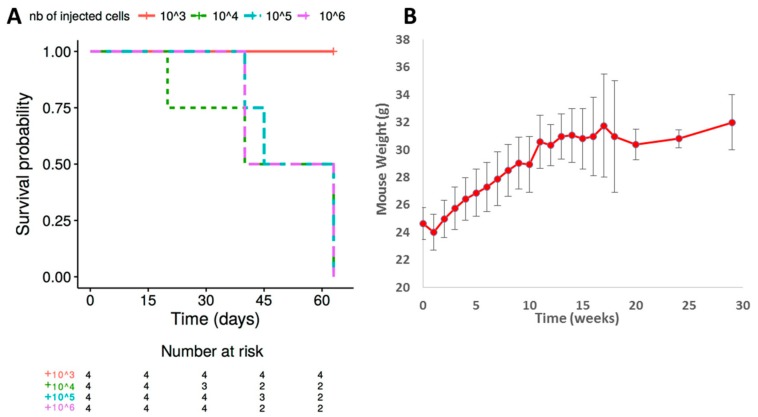
Follow-up of mice injected with L428-c cells. (**A**) Kaplan Meier presentation of mouse survival after injection of various numbers of cells. Mice injected with 10^3^ cells showed better tolerability and higher survival than those injected with higher numbers of cells. (**B**) Weight of mice injected with 10^3^ cells demonstrating a transient decrease of weight immediately after injection, followed by recovery. Thirty mice were used in this standardization process and evaluated weekly. Standard error bars are shown.

The first step to standardize the xenograft model was to determine the number of injected cells required to achieve tumor infiltration without side effects. A correlation between the number of cells injected (from 10^3^ to 10^6^ L428-c cells) and mortality was established (Figure 2A). All mice injected with 10^3^ cells survived for at least 28 weeks. In contrast, mice injected with 10^4^ to 10^6^ cells died within two months.

The second step was to determine the kinetics of tumor infiltration, an important standardization parameter for testing novel therapeutic molecules in animal models. A series of 30 mice, injected with 10^3^ L428-c cells, were monitored for 30 weeks. Morphological characterization, as well as an assessment of tumor infiltration, was performed weekly. We observed no significant decrease in the weight of the mice during the follow-up (Figure 2B) with no mortality.

**Figure 3:**
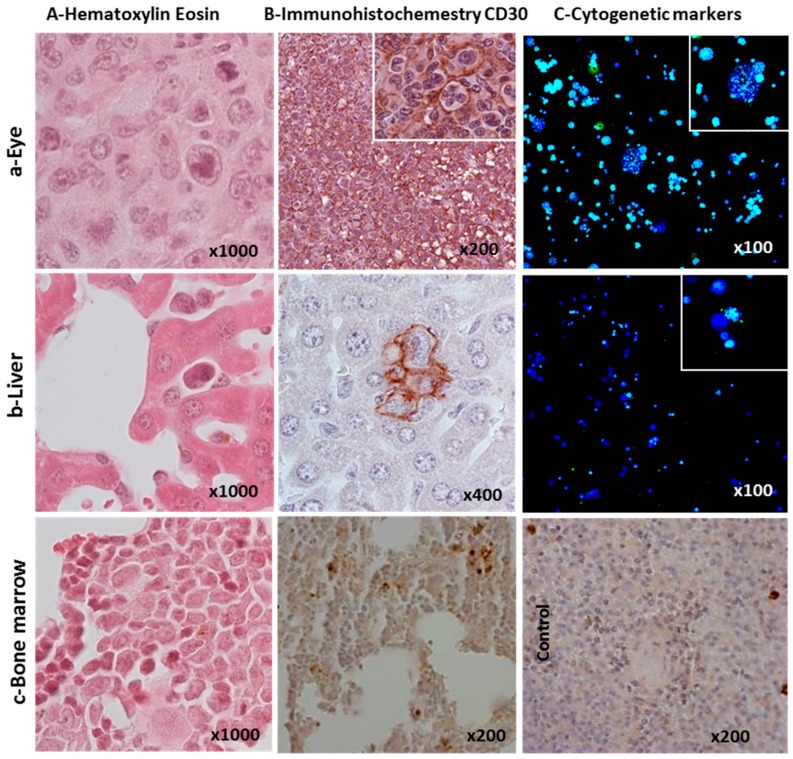
Organ infiltration and tumor markers identifying the L428-c subline in NOD/SCID/gamma (−/−) (NSG) mice. (**A**) Hematoxylin and eosin, (**B**) Immunohistochemical, and (**C**) Cytogenetic markers using human centromere probes (green) are shown. Infiltration of tumor cells in the (a) orbit/ocular tissues (site of injection), (b) the liver, and (c) bone marrow. Monkey spleen served as a positive control for CD30 staining.

Tumoral infiltration was evident and clearly detectable in the liver, bone marrow, and spleen from the seventh week after cell transplantation by flow cytometry, immunohistochemistry, immunofluorescence, and cytogenetic marker levels (Supplementary Table S2). Histological analyses by HE and CD30 staining of infiltrated organs of NSG mice revealed the presence of large multinucleated CD30+ cells (Figure 3A,B) in HL tumors in the various organs listed in Supplementary Table S2. Human-specific centromere sequences were identified in both large and small cells (Figure 3C).

**Figure 4:**
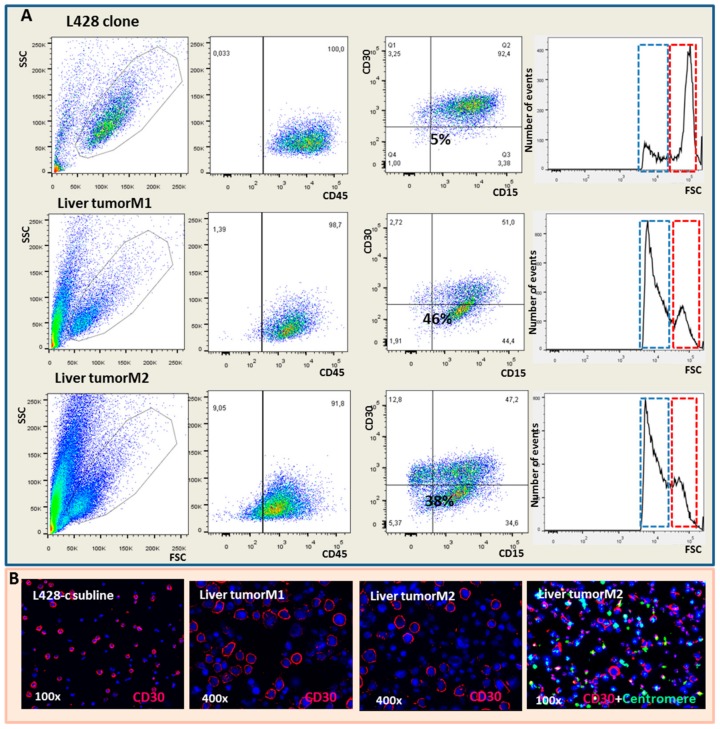
Analysis of the cells recovered from liver tumors of two mice (M1 and M2) 32 weeks after injection of 103 L428-c cells by flow cytometry, immunofluorescence microscopy, and human centromere staining. (**A**) flow cytometry analysis of liver tumor cells was performed using the surface marker CD45 to select for human cells and CD15/CD30 as specific markers for HL. The proportion of each sub-population with respect to CD30 and CD15 marker levels is presented. The FSC demonstrates the presence of two cell populations, in both the injected cells and in the liver tumor cells, although at different frequencies. The red box shows the mean FSC of injected cells and the blue box the mean FSC of HL cells recovered from liver tumors of NSG mice. Liver tumor cells contained a higher proportion of CD30−/CD15− cells (5% and 2% vs. 1% for L428-c and 2% for mouse 1) and CD30− (46% (M1) and 40% (M2) vs. 5% for L428-c). (**B**) Immunofluorescence staining for CD30, showing the presence of cells without CD30 staining in the L428-c subline and mouse liver tumors One of the panels shows immuno-FISH specific for CD30 and for human centromere sequences confirming the presence of a small population of CD30 negative human cells with a high intensity of centromere sequences in a mouse liver. HRS cells were CD30 positive and showed positive FISH signals specific for centromeres sequences.

Immunophenotype analysis by flow cytometry after CD30/CD15 staining confirmed the results obtained by immunohistochemistry and FISH (Supplementary Figure S4). A large proportion of the human cells in the mice were small and consisted of a large proportion of CD30− cells, ranging from 20% to 50% of the cells (Figure 4).

### 2.3. Cytogenetic Characterization of HL Cells Grown In Vitro and In Vivo

#### 2.3.1. Ploidy Deviations of In Vitro and In Vivo Growing HL Cells

**Figure 5:**
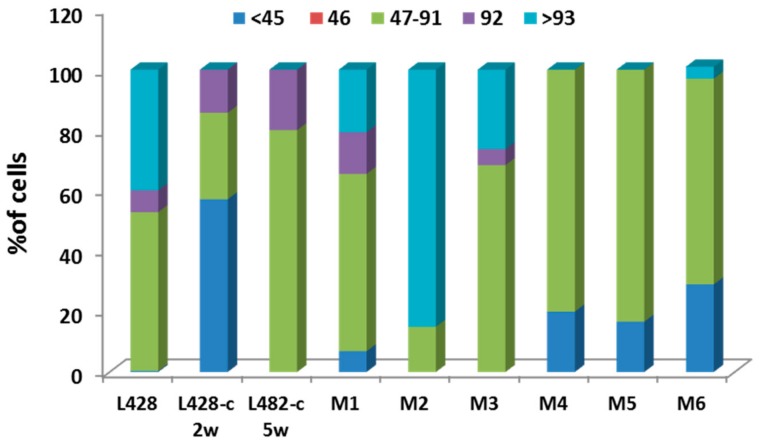
Numerical chromosomal aberrations were scored after telomere and centromere staining of the L428 parental cell line, the L428-c subline after two and five weeks in culture, and in HL cells derived from the livers of six mice (M1–M6) 16 and 32 weeks after injection, respectively.

We monitored putative changes in ploidy of the HL cells using FISH with specific human centromere probes and telomere sequences on expanded cell cultures after cloning in methylcellulose, followed by CD30−/CD15− cell sorting, and finally in vivo growth in mice. The proportion of small cells (containing < 46 chromosomes) among L428-c cells increased after two weeks of in vitro expansion. The proportion of cells with an abnormal number of chromosomes increased progressively throughout in vitro and in vivo cell expansion (Figure 5). In addition, the proportion of large cells increased with time in culture and was twice as high in late cultures than in early ones. Thus, the initially growing L428-c cells appear to be small cells that present abnormalities in the segregation of whole chromosomes and cell ploidy.

#### 2.3.2. Telomere Dysfunction

**Figure 6:**
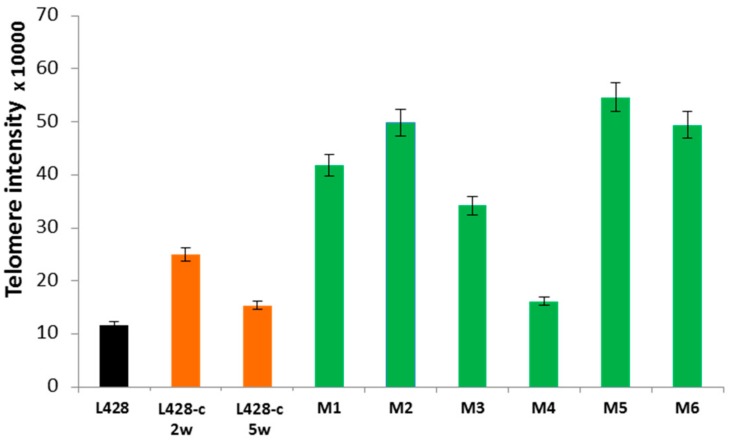
Comparison of telomere length in the L428 cell line, L428-c subline expanded in vitro for two or five weeks, and HL cells recovered 16 and 32 weeks, respectively, from the livers of mice (M1–M6). All experiments were performed in triplicate and 200 metaphases were scored. The mean ± SEM is shown.

Telomere dysfunction could be an important mechanism underlying genomic instability in HL. We first assessed the mean telomere length and telomere aberrations of HL cells grown in vitro and in vivo by Q-FISH, the technique of choice for high-resolution telomere-length quantification. Figure 6 shows the mean fluorescence intensity of telomeres in HL cells obtained from the parental L428 cell line, from L428-c cells after two and five weeks, respectively, of in vitro expansion and subsequent cell sorting, and liver tumor cells derived from the mice. Telomere length of L428-c and human cells recovered from the livers of mice were significantly greater than those of the parental L428 cell line (Figure 6). Of note, telomeres were significantly shorter in the L428-c subline after five weeks than after two weeks of expansion.

**Figure 7:**
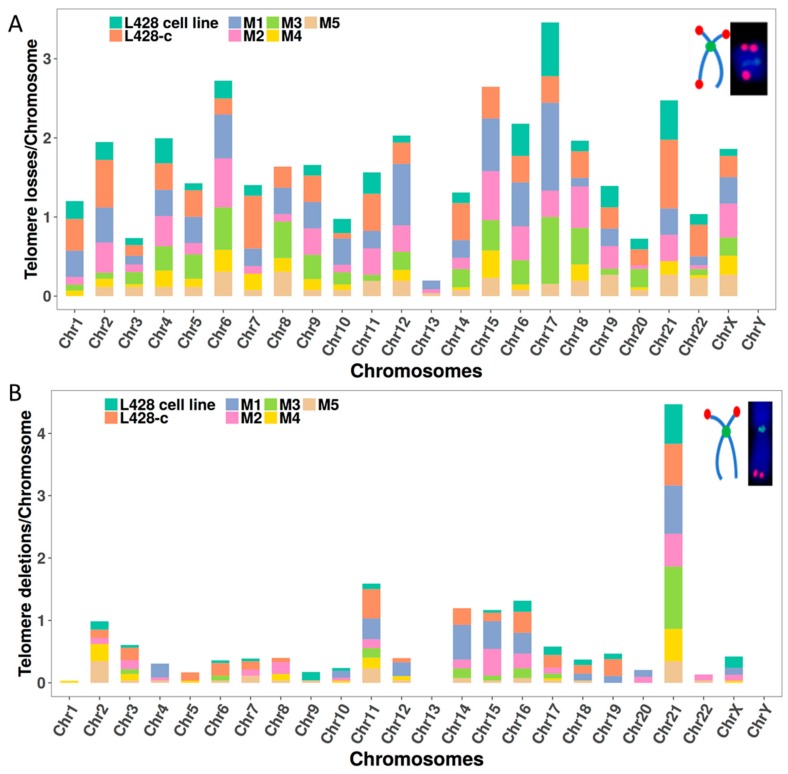
Telomere aberrations in each chromosome in the parental L428 cell line, L428-c subline, and in liver-derived HL cells from five mice (M1–M5). (**A**) Telomere loss and (**B**) Telomere deletion assessed on the basis of telomere and centromere staining followed by M-FISH. For technical reasons telomere aberration analysis was not performed on cells from mouse 6.

We then scored telomere aberrations, such as the loss of one telomere or two telomeres in the same arms, called telomere deletion. There was a substantially higher incidence of telomere loss in cells growing in vitro and in HL cells recovered from the livers of the NSG mice than that in the parental cell line (Figure 7A). Importantly, we observed a recurrent high frequency of telomere loss from the same chromosomes in all analyzed cells, irrespective of their origin.

Similarly, the frequency of telomere deletion was high in cells cultured in vitro and in those from HL tumors in the livers of NSG mice (Figure 7B). Telomere deletions involved chromosomes 2, 11, 14, 15, 16, and 21 in all analyzed cells.

Thus, the second cytogenetic characteristic of HL cells grown in vivo or in vitro appears to be telomere dysfunction.

#### 2.3.3. High Incidence of Dicentric Chromosome and Centric Ring Formation in HL Cells

**Figure 8:**
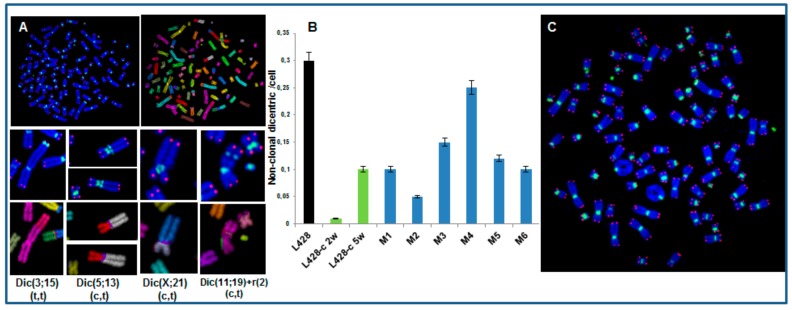
Dicentric chromosomes and centric rings in HL cells. (**A**) Clonal dicentric chromosomes in L428 cells, L428-c cells, and HL cells recovered from mouse liver. The dicentric chromosomes are characterized by telomere (t,t) and/or centromere (c,t) breakpoints detected by telomere and centromere staining followed by M-FISH. (**B**) Frequency of non-clonal dicentric chromosomes in parental L428 cells, cells of the L428-c subline after two and five weeks of expansion, and HL cells recovered from mouse livers (M1–M6). (**C**) Representative image of a metaphase with a non-clonal ring of chromosome 4 related to telomere fusion in an HL cell from mouse liver (63× magnification).

We used telomere and centromere staining followed by M-FISH to score the frequency of dicentric and centric ring chromosomes in the parental cell line, in cells growing in vitro and in HL cells isolated from mice. Clonal dicentric chromosomes were found in all analyzed metaphases involving chromosomes 3 and 15 (dic(3;15)), 11 and 19(dic(11;19)), 5 and 13 (dic(5;13)), and 21 and X (dic(X;21)). Similarly, small centric rings of chromosome 2 were observed in all metaphases, resulting from breakpoints near the centromere (Figure 8A). Additional dicentric chromosomes and rings were observed in all HL cells grown in vitro, as well as in vivo (Figure 8B). M-FISH analysis revealed that the dicentric chromosomes were the result of chromosome fusion, and interstitial telomeres were observed for some dicentric chromosomes (Figure 8A–C). Overall, the dicentric chromosomes and centric ring chromosomes identified in HL cells were characterized by breakpoints near the centromeric or telomeric regions.

Thus, the third characteristic of cells grown in vitro and in vivo is a high frequency of dicentric chromosomes and centric rings ascribed to telomere uncapping and /or centromere instability.

#### 2.3.4. Karyotype of Cells Grown In Vitro and In Vivo

The numerical and structural chromosomal aberrations identified in cells after in vitro expansion and those grown in vivo in mice were scored in analyses of at least 30 metaphases.

**Figure 9:**
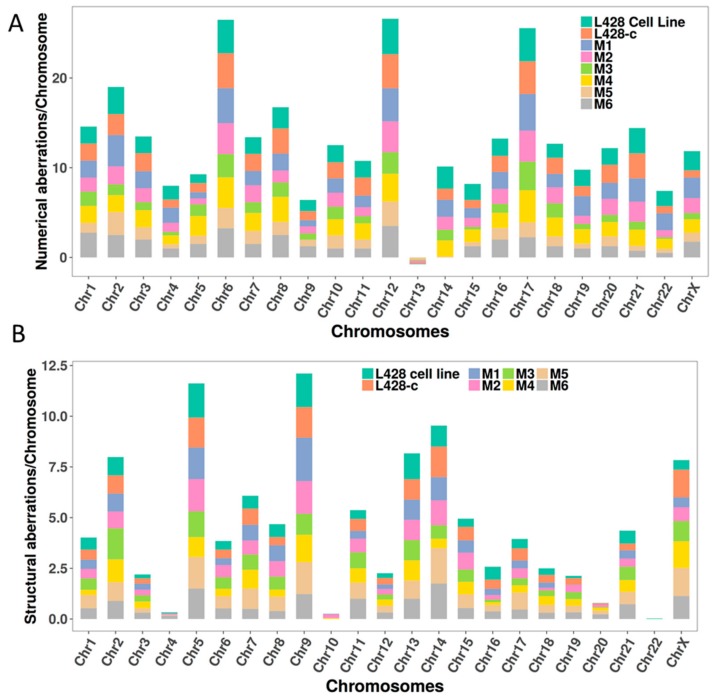
Chromosomal aberrations detected by telomere and centromere staining followed by M-FISH in the L428 cell line, L428-c subline, and HL cells derived from the livers of different mice (M1–M6). (**A**) Numerical aberrations (gains and losses). (**B**) Structural chromosome aberrations, including translocations, insertions, deletions, and dicentric, ring, and acentric chromosomes. Thirty metaphases were analyzed, and the chromosomes classified from each cell line.

The frequency of numerical aberrations in each chromosome of L428 cells, L428-c cells, and HL cells derived from mouse livers are shown in Figure 9A. The parental L428 cell line has the highest frequency of chromosomal rearrangements compared to cloned cells grown in vitro and in vivo (Figure 9B). Importantly, the same chromosomes involved in the chromosomal rearrangements were observed in the parental cell line as well as in vitro and in vivo expanded cells, thus demonstrating the unique and clonal origin of all cells analyzed.

**Figure 10:**
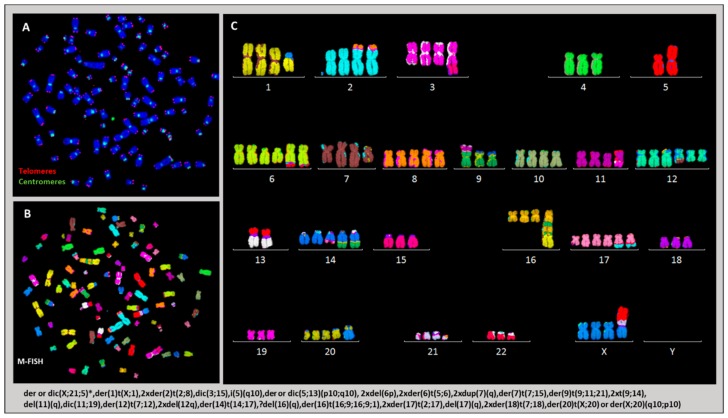
Chromosomal rearrangements in L428 cells. (**A**) Telomere and centromere staining followed by (**B**) M-FISH allows (**C**) detailed classification of chromosomes and identification of all clonal chromosomal aberrations listed at the bottom of the figure. * Only the dic(X;21) is clonal, and the tric(X;21;5) is an additional aberration (63× magnification).

We used telomere and centromere staining to successfully establish, for the first time, a precise classification of chromosomal rearrangements and to determine several previously undefined markers in the L428 cell line (Figure 10) [21]. The mean number of chromosomes per metaphase was 94 (71–103). We found a complex karyotype, marked by the presence of clonal dicentric chromosomes and isochromosomes: (dic(3;15), dic(5;13), dic(11;19) and dic(X,21) and i(5q)). In addition, we detected aberrations resulting from BFB cycles, such as der(16)t(16;9;16;9;1). Such aneuploidy was associated with a high frequency of chromosomal aberrations and high frequency of non-clonal dicentric chromosomes.

**Figure 11:**
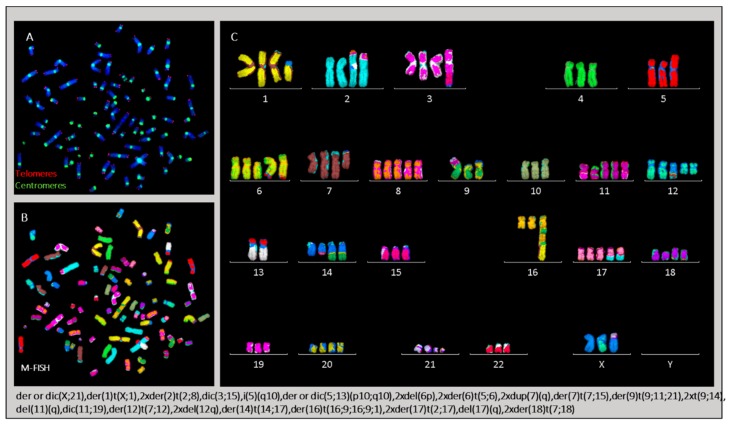
Chromosomal aberrations detected in L428-c cells by telomere and centromere staining (**A**) followed by M-FISH (**B**,**C**). Most clonal aberrations listed at the bottom of the figure are shared with those in the parental L428 cell line (63× magnification).

Similarly, the L428-c subline presented numerical chromosomal aberrations after two weeks of expansion, with the presence of small metaphases. The mean number of chromosomes per metaphase was 47 (19–93). We also detected clonal dicentric chromosomes, as well as a complex karyotype. We found the same chromosomal aberration profile in CD30−/CD15− cells sorted after two weeks of expansion. During in vitro expansion, the L428-c subline and CD30−/CD15− cells acquired a chromosomal phenotype very similar to that of the L428 parental cell line with mean number of chromosomes being 87 (74–92) (Figure 11).

**Figure 12:**
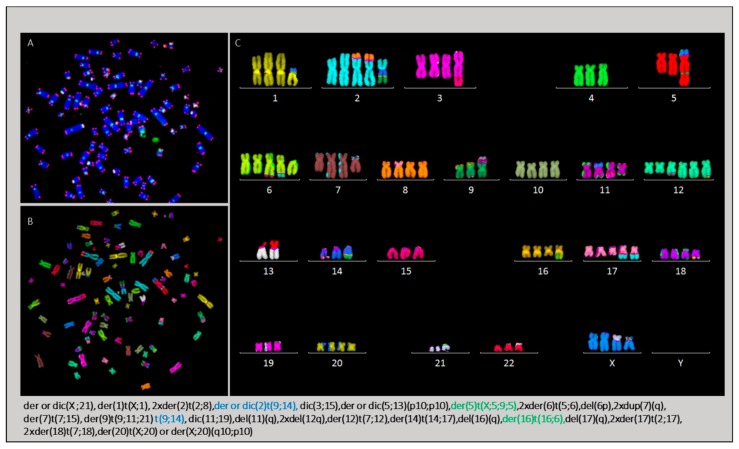
Cytogenetic characterization of HL cells recovered from a mouse liver by (**A**) telomere and centromere staining followed by (**B**) M-FISH. This approach allowed a detailed classification of chromosomes (**C**). Clonal chromosomal aberrations are listed and specific aberrations compared to those of the L428 cell line and L428-c subline are highlighted in green and blue, respectively, at the bottom of the figure (63× magnification).

The karyotype of HL cells recovered from the livers of mice presented numerical and structural chromosomal aberrations largely similar to those identified in L428 and L428-c cells. The mean number of chromosomes varied between 71 (45–88) and 92(84–97). A complex karyotype was found (Figure 12).

**Figure 13:**
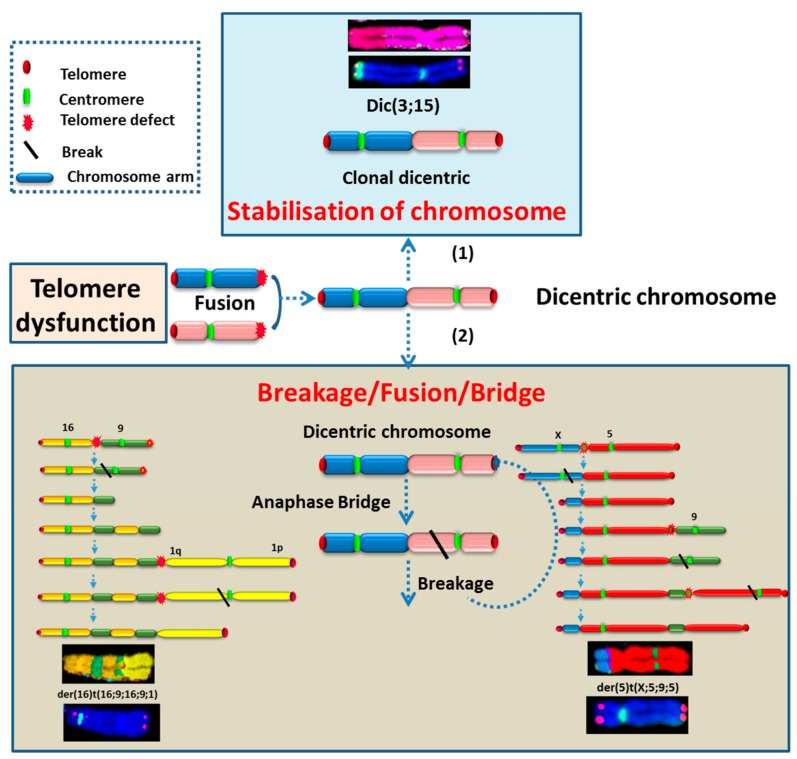
Schematic representation of genome rearrangements induced by telomere dysfunction and dicentric chromosome formation: (1) stabilization of dicentric chromosome and the formation of clonal dicentric, for example dic(3;15) (2) B/F/B cycles and the putative steps for the derivation of the der(16)t(16;9;16;9;1) and der(5)t(X;5;9;5) via B/F/B cycles were represented.

The specific clonal chromosomal aberrations identified in in vivo growing cells were typically initiated by BFB cycles, leading to the formation of non-clonal dicentric chromosomes prone to other rearrangements, such as der(5)t(X;5;9;5) and der or dic(X;20) (Figure 13). This aberration was also detected in L428-c cells, although in only very few cells, suggesting strong selection in the mouse (Supplementary Figure S5).

#### 2.3.5. The Occurrence of Chromosomes Involved in Chromosomal Aberrations Correlates with Their Telomere Dysfunction Profile

**Figure 14:**
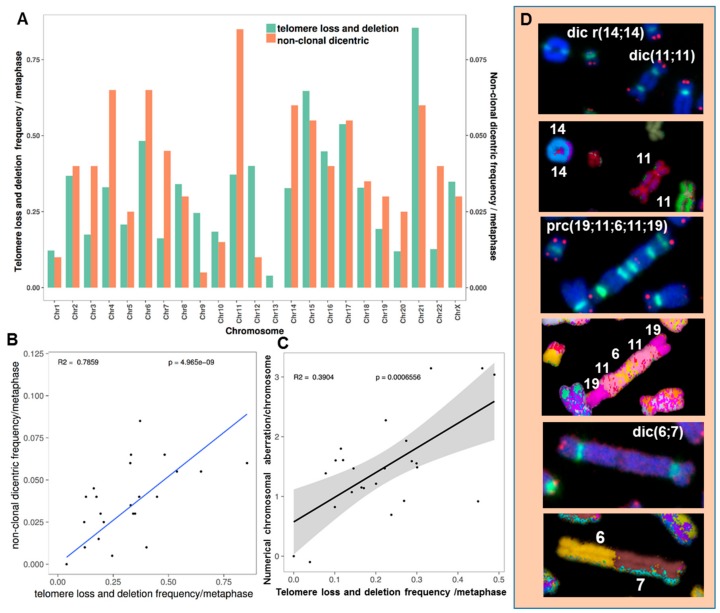
Chromosomal instability in HL cells correlates with telomere dysfunction. (**A**) The distribution of individual chromosomes involved in non-clonal dicentric chromosome formation correlates with the profile of individual chromosomes with telomere dysfunction (loss and deletion). The diagram represents all HL metaphases analyzed. (**B**) Regression analysis between the frequency of telomere loss among the different chromosomes and their involvement in non-clonal dicentric chromosomes. The diagram represents all HL metaphases analyzed. (**C**) Regression analysis between the frequency of telomere loss among the different chromosomes and their involvement in numerical chromosomal aberrations. (**D**) Partial metaphases showing non-clonal dicentric and ring chromosomes. Interstitial telomeres were detected at the breakpoint, suggesting that dicentric chromosome formation is related to telomere dysfunction (63× magnification).

We explored a putative correlation between telomere dysfunction and dicentric chromosome formation by first examining telomere dysfunction in metaphases (deletion and loss) of HL cells and all chromosomal rearrangements. No correlation between structural chromosomal aberrations and telomere dysfunction was identified. Importantly, there was a close relationship between the occurrence of non-clonal dicentric chromosomes and telomere loss and telomere deletion (*p* < 10^−8^) in HL cells derived from in vitro as well as in vivo expansion (Figure 14A,B). In addition, there was a significant correlation between telomere loss and numerical chromosomal aberrations (*p* < 10^−3^) (Figure 14C).

#### 2.3.6. Telomere Maintenance of HL Cells Grown In Vitro and In Vivo

**Figure 15:**
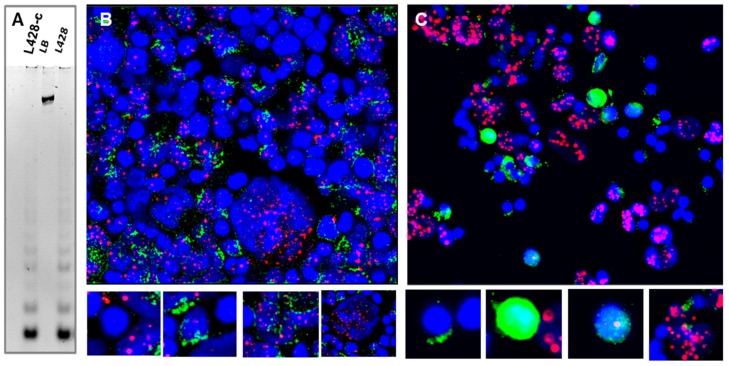
Telomerase expression in HL cells grown in vitro and in vivo. (**A**) High telomerase activity detected in the L428-c subline relative to that of the parental cell line (L428). Lysis buffer (LB) served as an internal control for the amplification, excluding false negatives. (**B**) Immunofluorescent staining of hTERT (green) and PML (red) demonstrates high telomerase expression in small cells of L428-c, as well as the presence of cells expressing both hTERT and PML. There are also large cells with a morphology similar to that of HRS cells, with very low hTERT expression and a large number of PML bodies. (**C**) Populations of HL cells recovered from the livers of mice included small cells with high telomerase expression, cells expressing both hTERT and PML, and large cells with a large number of PML bodies. We also detected cells with no expression of hTERT or PML among L428-c cells and HL cells recovered from the livers of mice (40× magnification).

We assessed telomerase activity in the L428 cell line and the L428-c subline by the Telomerase Repeated Amplification Protocol (TRAP) assay. L428-c cells exhibited higher telomerase activity than the parental L428 cells (Figure 15A). We confirmed these results by co-immunofluorescence of hTERT associated with promyelocytic leukemia (PML) (Figure 15B). Interestingly, small cells exhibited higher telomerase expression than HRS cells. PML bodies were found in HRS cells and correlated with no or with very low telomerase expression. Telomerase expression in HL cells derived from mice was assessed by immunofluorescence analysis only. Small HL cells recovered from mice five weeks after transplantation also had high levels of telomerase expression (Supplementary Figure S6). After 16 weeks of in vivo expansion of the HL cells, we observed small cells with high hTERT expression and HRS-like large cells that expressed low or no hTERT, but contained more PML bodies (Figure 15C). Similar observations were made after 32 weeks of in vivo expansion.

Thus, we conclude that the HL cells that primarily grow in vitro, as well as in vivo, are characterized by high telomerase expression.

### 2.4. Telomerase Activity and Telomere Dysfunction in Circulating Lymphocytes of HL Patients

**Figure 16:**
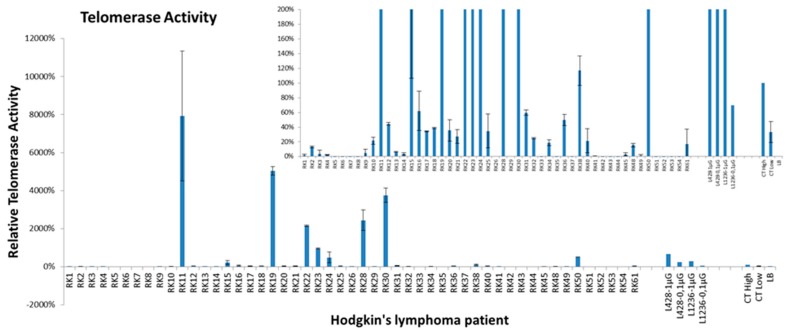
Detection of high telomerase activity in circulating lymphocytes of ten HL patients using the repeat amplification protocol (TRAP). Histogram displaying the fold change of relative telomerase activity in HL patients relative to that of CT (positive control equal to 100%). Telomerase activity of the L428 and L1236 HL cell lines served as a second control. The experiments were performed in triplicate.

We extended our findings on the high telomerase expression and on telomere dysfunction in HL progenitors derived from cell lines by determining telomerase activity and assessing telomere dysfunction in circulating lymphocytes from 50 HL patients and 50 healthy donors. We detected high telomerase activity in HL cells from ten of the patients, but none in cells from any of the healthy donors (Figure 16).

**Figure 17:**
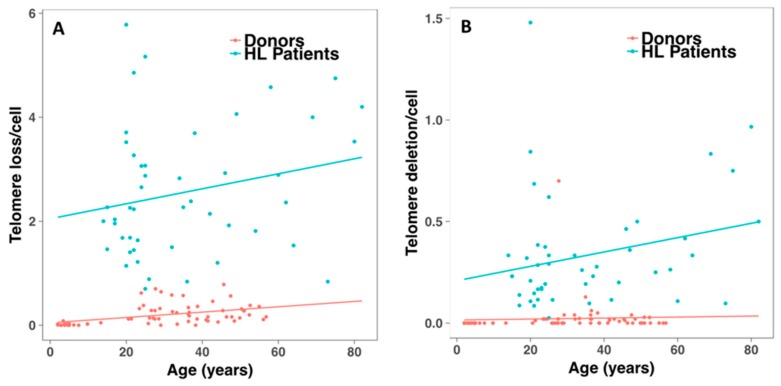
Telomere dysfunction detected in circulating lymphocytes of HL patients. (**A**) Significant difference between the frequencies of telomere loss for HL patients and those detected for healthy donors. (**B**) The same results were observed for telomere deletion. Telomere deletion and telomere loss in lymphocytes of healthy donors are represented by the red circles and in those of HL patients by blue circles, respectively.

Telomere shortening in these patients has previously been described [22]. Here, we observed greater telomere loss and a higher frequency of telomere deletions in circulating lymphocytes of HL patients than in those of healthy donors (Figure 17). Telomere loss and deletion did not correlate with age, and there was high interindividual variation (*p* = 0.88, *R*^2^ = 0.007 and *p* = 0.99, *R*^2^ < 10^−7^). Nevertheless, there was a significant correlation between telomere loss and age for healthy donors, with high interindividual variation (*p* < 10^−3^, *R*^2^ = 0.042).

### 2.5. Preclinical Effect of a First In-Class Alkylating HDAC Inhibitor Fusion Molecule (EDO-S101) in the HL Animal Model

Having established a reproducible and robust animal model for HL, we wished to validate its potential for exploring novel drugs for the treatment of HL. We chose a well-known drug for the treatment of HL, namely, EDO-S101.

EDO-S101 (Tinostamustine) is a first-in-class alkylating deacetylase inhibitor (AK-DACi) molecule that fuses the DNA damaging effect of Bendamustine, an active salvage regimen for patients, with the fully functional pan-histone deacetylase (HDAC) inhibitor, Vorinostat, in a completely new chemical entity [23]. In addition, Vorinostat is one of the HDAC inhibitors with a favorable immune modulatory effect [24,25]

**Figure 18:**
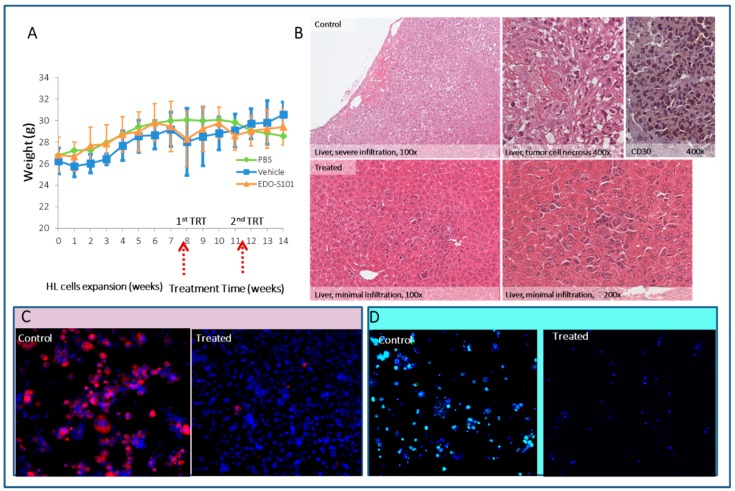
In vivo administration of two successive EDO-S101 doses (60 mg/mL). (**A**) Decrease of weight of mice after each injection of EDO-S101 and of vehicle, after which, weight was regained. (**B**) Tumor cell infiltration in the livers of control and of treated mice demonstrated by immunohistochemistry after (**B**) H&E, (**C**) CD30, and (**D**) human centromere staining (green signal) after three weeks of treatment. Thirty mice were treated with two successive EDO doses seven weeks after HL cell transplantation. Mice were sacrificed seven weeks after EDO treatment (10× magnification).

First, we treated seven HL cell lines in vitro with EDO-S101 alone, or in combination with irradiation, and demonstrated its high toxicity with IC50s ranging from 1.6 to 6.3 µM (Supplementary Figures S7 and S8). Next, we assessed the effect of EDO-S101 in our HL animal model. Seven weeks after transplantation of HL cells, the animals were subjected to a single injection (60 mg/kg or 80 mg/kg) or to two injections of 60 mg/kg at an interval of three weeks. EDO-S101 demonstrated excellent tolerance based on weight stability (Figure 18A). Histological examination of the animals after three weeks of treatment demonstrated a significant decrease of tumor infiltration and the absence of necrotic cells and cellular degeneration in treated mice (Figure 18B). Treatment with a single dose of 80 mg/kg EDO-S101 was more effective than injecting two successive doses of 60 mg/kg. Mice that received 2 × 60 mg/kg exhibited some infiltration of HL cells, and no tumor cells were detected at doses of 80 mg/kg (Figure 18B–D).

## 3. Discussion

Studies on mechanisms leading to malignant transformation of B cells into HL are hampered by the lack of a reproducible animal model. Such models are also to be used for the search for more effective and less toxic treatments of the approximately 20% of patients who relapse or become refractory to treatment. The only validated animal model of HL currently being used was established from a derivative of HL cell line L540, which is of T-cell origin [9].

Here, we report the establishment of a highly efficient and robust HL animal model. We successfully transplanted L428-c cells derived from the L428 HL cell line into total body irradiated NSG mice. Injection of as few as 10^3^ cells resulted in engraft and tumor development in vivo. The resulting animal model shows high survival and low toxicity from the tumor. Moreover, the model is suitable for testing novel drugs for the treatment of HL, as demonstrated by the effect of EDO-S101 on tumor progression. Our model contributes to our understanding of characteristics of HL cells engrafted in NSG mice, with emphasis on the cytogenetic events and steps during their transformation into HRS cells [21].

The presence of initiating clonotypic cells or “cancer stem” cells in classical HL has been a subject of debate for at least one decade [13,17]. Indeed, the likelihood that morphologically normal B cells are responsible for the generation of HRS cells has been well documented [12,14,15].

Faced with the difficulty of isolating clonotypic B cells from HL patients by fluorescence-activated cell sorting [13], the rarity of these cells and their low or inexistent capacity to proliferate in vitro in the absence of their micro-environment [13], we sought to determine appropriate experimental culture conditions for growing and amplifying such cells before transplanting them into NSG mice. We assessed the clonogenicity of seven HL cell lines. Only three of the seven HL cell lines, L428, KMH2 and L540, gave rise to clones in semi-solid medium (methylcellulose), although at varying efficiencies, with L428 cells being the most clonogenic. One of the clones, the L428-c subline, initially exhibited a low rate of proliferation that increased over time in culture. L428-c cells had higher cloning efficiency than the parental L428 cells. These results confirm and extend previous reports by Newcom and Jones using the L428 cell line [12,13]. Interestingly, immunophenotypic analysis of L428 clones (L428-c) revealed a high frequency of CD30−/CD15− cells that contained a high proportion of mononucleated cells compared to parental cells. Furthermore, we demonstrate the capacity of fluorescence-activated cell sorted CD30−/CD15− HL cells to develop into cells with an immune-phenotype similar to that of the L428-c subline, with high clonogenicity and in vitro proliferation. Cytogenetic analyses clearly demonstrated a higher frequency of metaphases containing less than 46 chromosomes in L428-c than that observed in the parental cell line. In addition, these cells displayed similar cytogenetic changes involving both numerical and structural aberrations. Based on these unique cytogenetic characteristics, we conclude that these sublines arose from single cells, i.e., that they are of clonal origin.

The high clonogenic efficiency of the L428-c subline allowed transplantation of these cells into immune-deficient NSG mice after total body irradiation using only 10^3^ cells. We observed tumor infiltration, essentially in liver, bone marrow, spleen, and brain, in addition to the eye when this was the injection site. Our data confirm and extend previous results from a single report on the attempt to obtain an HL animal model using the L428 cell line. However, that study used 2 × 10^6^ L428 cells injected into unirradiated NSG mice [3]. All other reported attempts to obtain an HL animal model using the L428 cell line have been unsuccessful [4]. Immunophenotypic characterization of HL cells recovered from mice clearly demonstrates a high frequency of small cells lacking the classical HL surface markers CD30 and CD15.

The phenotypic transition acquiring more classical HL markers was associated with a shift towards aneuploidy and accumulation of chromosomal aberrations. Cytogenetic characterization of cells grown in vitro and in vivo show that: (1) the tumor cells are derived from diploid cells or small cells; (2) aneuploidy is an inherent characteristic of multiplying HL cells; (3) telomere dysfunction appears to be a major cause of chromosomal aberrations in these small cells and is associated with high telomerase activity; and (4) the formation of dicentric chromosomes and centric rings in HL cells is related to chromosome fusion due to telomere uncapping and represents the first step in the generation of cytological genomic instability observed in HL via breakage/fusion/bridge (B/F/B) cycles [26,27]. In-keeping with a telomere-dependent origin of chromosomal instability, we found that the distribution of chromosomes involved in non-clonal dicentric chromosomes and centric rings correlated with the profile of telomere dysfunction. Most importantly, the chromosomal aberrations, including dicentric chromosomes, in the L428-c subline and HL cells recovered from mouse livers were very similar to those detected in the L428 cell line. We have previously demonstrated that these dicentric chromosomes correlate with telomere loss or deletion and chromosome fusion. The presence of sub-telomeric sequences in the dicentric chromosome breakpoints was demonstrated previously in the dic(3;15) [28]. The difference observed between L428-c cells maintained in vitro and in vivo compared to the L428 cell line was related first to the high incidence of non-clonal dicentric chromosomes and centric rings and secondly to the aberrations induced by B/F/B cycles related to the formation of these non-clonal dicentric chromosomes. For example, der(16)t(16;9;16;9;1) with i(5q) was detected in L428 cells, L428-c cells, and HL cells from the liver of one mouse. Furthermore, der(5)t(X;5;9;5) was detected in livers from five mice. This aberration was found in the L428-c subline, but it was not clonal. Importantly, there was a significant correlation between numerical chromosomal aberrations and telomere loss for each chromosome. We have previously demonstrated that most of the micronuclei in L428 cells contain centromere sequences related to chromosome lagging at mitosis [28]. Consistent with these results, we speculate that telomere loss can induce dicentric chromosome formation or micronucleus formation as well as chromosome lagging. These data extend and underscore our previous findings concerning the implication of telomere dysfunction in HL chromosomal instability via B/F/B cycles and micronucleus formation [28,29]. Of note, we provide in this study the first complete karyotype of the L428 cell line and demonstrate the feasibility of cytogenetic analysis in a xenograft HL model.

The telomere dysfunction observed in HL cells requires the activation of a telomere maintenance mechanism to support immortalization. L428-c cells had higher telomerase activity than the parental L428 cells. Immunofluorescence revealed higher telomerase expression in small than large HRS-like cells, characterized by higher numbers of PML bodies. Similarly, we found high hTERT expression in small cells derived from in vivo tumor expansion. In contrast, HRS cells exhibited very low or no hTERT expression, but contained a greater number of PML bodies. In addition, co-localization of TRF2 and PML suggests that alternative lengthening of telomeres (ALT) mechanisms are involved in telomere maintenance in HRS cells. Taken together, these data confirm our previous study concerning the coexistence of both telomerase and ALT telomere maintenance mechanisms in HL cells [30]. Moreover, we demonstrate that the early HL cells detected in vivo exhibited higher telomerase expression suggesting that they may represent the “cancer stem cells” of HL [31]. Consistent with this possibility, we found high telomerase activity associated with telomere dysfunction [22] in circulating lymphocytes of a subset of HL patients (10/50 patients). In addition, the frequency of telomere loss and deletion was significantly higher in circulating lymphocytes of HL patients than in those of healthy donors, and it was not age dependent. We previously demonstrated a higher frequency of dicentric chromosomes in circulating lymphocytes of HL patients prior to any treatment [32]. It may be informative to investigate telomere dysfunction in HL families to assess the role of genetic susceptibility and a possible second event in this disease, such as viral infection [33,34]. Recently, a germinal mutation in *POT1* in two HL families was detected [35].

We evaluated the antitumor effect of EDO-S101 in NSG mice xenografted with L428-c cells. We observed high in vitro sensitivity of seven HL cells lines after EDO-S101 treatment on its own or combined with radiation therapy. In our animal model, histological evaluation of infiltrating HL cells after EDO-S101 treatment showed a significant decrease of HL cell infiltration and lack of necrotic cells [36]. In this context should also be mentioned that, we were unable to obtain human metaphases from cells recovered from mice as well as from prolonged cultures of HL cells recovered from EDO-treated mice. The effect of EDO-S101 was more prominent in those small cells that exhibited high telomerase expression than HRS-like cells. We speculate that EDO-S101 blocked the transition from the telomerase to ALT profile as described previously [30]. It will be important to test EDO-S101 in other models that exhibit the switch between telomerase and ALT mechanisms, as well as models characterized by high telomerase activity.

Here, we propose novel in vitro strategies for the selection and expansion of HL cells for transplantation into NSG mice. It may be informative to directly re-transplant CD30− HL cells recovered from liver tumors that are derived from the L428-c subline. Molecular characterization of these cells as well as CD30+ HL cells may shed light on possible mechanisms and steps in the transformation of these negative cells to CD30+ cells. Interestingly, attempts to apply the same approach using cells derived and expanded from circulating lymphocytes or tumor tissues from relapsed/refractory HL patients should be carried out. We have also reported that the tumor initiating cells are characterized by high telomere dysfunction and high telomerase activity. Similar findings have been made on circulating lymphocytes from HL patients and could explain the absence of unique and recurrent translocations in HL [37].

## 4. Materials and Methods

### 4.1. Materials

#### 4.1.1. Cell Lines, Patient Samples and Cell Cultures

The human HL-derived cell lines L428 KMH2, L591, HDLM2, L540, SUP-HD, and L1236 were cultured in RPMI 1640 medium supplemented with Glutamax (Gibco-BRL, Grand Island, NY, USA), 10%FBS (Eurobio, Courtaboeuf, France) and antibiotics (Gibco-BRL) at 37 °C. Peripheral blood lymphocytes were obtained from 50 patients with HL before treatment and from 100 healthy donors, who granted informed consent and approved by the Gustave Roussy Institutes Institutional review (Table 1).

Mononuclear cells from blood were isolated by density centrifugation (Ficoll-Paque; Life Science) and kept at −80 °C. Cytogenetic preparations were made from HL patients and healthy donors [22].

The collection of blood samples from patients and donors was approved by the Ethics Committee of Gustave Roussy Cancer Campus University Paris Saclay: approval number 97-06. All donors and patients have signed an informed consent form consistent with institutional review board guidelines.

#### 4.1.2. Xenograft Models

Immunodeficient NOD-SCID-gammac−/− (NSG) mice aged 5 to 6 weeks, were purchased from Charles River (NOD.Cg-Prkdcscid Il2rgtm1Wjl/SzJ, Saint-Germain-Nuelles, France). The mice arrived two weeks prior to initiating the experiments and were housed in micro-isolator cages during the entire course of the study. Immediately prior to the injection of HL cells, 60 mice were exposed to 3 Gy gamma irradiation using an IBL637 ^137^Cs irradiator at a dose rate of 0.61 Gy/min. From 10^3^ to 10^6^ cells, either from the L428, L428-c, KMH2, L591 and L1236 cell lines, were injected intravenously via the retro-orbital sinus or the tail.

The mice were killed by cervical dislocation and various organs were analyzed and tumoral infiltration examined using multiple techniques.

#### 4.1.3. In Vivo EDO-S101 Activity

Tinostamustine (EDO-S101) was provided by Mundipharma EDO-GmbH (Basel, Switzerland). The antitumoral effect of EDO-S101 was assessed in 50 NSG mice for 30 weeks and based on organ tumor cell infiltration and survival. One single dose (60 mg/kg or 80 mg/kg) (20 mice) or a repeat-dose (60 mg/kg) (30 mice) of EDO-S101 were injected at three-week intervals

### 4.2. Methods

#### 4.2.1. Fluorescence-Activated Cell Sorting and Flow Cytometry Analyses

Cells were gated to exclude apoptotic or necrotic cells and of CD30−/CD15−, CD30+ and CD15+/CD30+ populations were sorted by gating on the lowest and highest 5% PE-expressing cells, respectively. Following sorting, the CD30−/CD15− cell fractions were re-analyzed using a FACScan flow cytometer (Becton Dickinson, Franklin Lakes, NJ, USA) and found to be more than 98% pure. For phenotypic analyses of cell lines or sorted cells, cells were prepared as described, and then stained with mouse anti-human CD30-phycoerthrin (PE), CD15-fluorescein isothyocyanate (FITC) and CD45-APC (PE) (all antibodies from BD PharMingen, San Diego, CA, USA). Cells were subsequently analyzed using a FACS LSRII (Becton Dickinson). The parental HL cells served as a control for all experiments.

To distinguish human and murine cells, mouse anti-human CD45 was used in FACS analyses. The characterization of these selected cells was established using anti-human CD15 and CD30 antibodies. For each sample, 10,000 events were analyzed if possible. Cells without staining were used to control and fix the gating strategy. Each analysis included a negative control, as well as the expression of CD30 alone, CD15 alone, and CD15 and CD30 together. Only co-staining of CD30 and CD15 is shown (Supplementary Figure S9).

#### 4.2.2. Clonogenic Assays

To assess the clonogenic potential of the HL cell lines and sorted cells after in vitro expansion, between 10^3^ to 10^5^ cells, depending on their growth rate, were plated in 1 mL 1.2% methylcellulose (Stem cell Technologies, Grenoble, France) in RPMI 1640 medium supplemented with Glutamax containing 10% FCS (GIBCO-BRL) and 1% Hepes (GIBCO-BRL). Colonies consisting of at least 50 cells were scored approximately 14 days after plating using an inverted microscope. The clonogenic index was established and various cell surface markers were subsequently analyzed to establish the immunophenotype. Three independent experiments were performed in triplicate.

#### 4.2.3. Tissue and Slide Preparation

Following dissection of various organs, including tumors, one portion of cells was fixed in 4% paraformaldehyde (PFA) for Immunohistochemistry (IHC) while a second portion was used for FACS and cytogenetic analyses. To obtain single cell suspensions, the tissues were first cut into pieces. The cells were then physically disaggregated using an 80 µm nylon cell strainer and filtered a second time through a 70 µm nylon cell strainer (BD Biosciences, Erembodegem, Belgium). The injected cell line served as a control for all experiments.

#### 4.2.4. Immunohistochemistry Analysis

Organs were fixed in 4% PFA, trimmed, and post-fixed in 70% ethanol. They were then briefly processed using a vacuum inclusion processor and paraffin blocks were prepared. Five micrometer-thick sections were cut from these blocks and the resulting sections were stained with hematoxylin-eosin (H&E) for histopathological analysis. In addition, IHC was carried out on entire liver and spleen specimens to monitor the spreading of engrafted cells. The search for single cells or small groups of grafted HL cells was performed using an anti-CD30 antibody (DAKO, France; 1:40, EDTA pH9 pre-treatment) and the Ventana Discovery XT IHC system. Apoptosis and tumor necrosis were assessed after immunohistochemistry.

All IHC-stained sections were initially evaluated and scored by hematopathologists. The same slides and the original scores were subsequently reviewed by pathologist’s specializing in HL.

#### 4.2.5. Immunofluorescence to Assess CD30 Expression

Cells were cytospun onto poly-l-lysine-coated glass slides at 700 rpm for 4 min, fixed with 10% formalin for 10 min, and subsequently treated with 0.25% Triton X-100 solution for 10 min. After blocking with 5% bovine serum albumin (Sigma, France), the cells were incubated with an anti-CD30 antibody (DAKO A/S, Glostrup, Denmark) and subsequently treated with Cyanine 3 labelled anti-mouse IgG (Invitrogen, Carlsbad, CA, USA). As a negative control, staining was carried out in the absence of primary antibody. The injected cells served as a positive control.

Immuno-FISH was performed in order to the detect the colocalization of CD30 expression and human centromere sequences (Eurogentec, Liége, Belgique).

#### 4.2.6. Cytogenetic Analysis

Liver-derived HL cells from mice were cultured in the presence of RPMI 1640 supplemented with 10% FCS and antibiotics. Colcemid (0.1 µg/mL) was added 2 h before harvesting, and slides with metaphase chromosomes were prepared following the standard methanol/acetic acid (3/1, *v/v*) procedure [32].

Six new liver-derived HL cell lines were generated after in vitro expansion. Cytogenetic analysis was performed first using telomere and centromere staining with PNA probes ((Eurogentec, Liége, Belgique) to quantify telomere length and telomere aberrations and detect chromosomal aberrations [38]. Next, karyotype analysis was performed on the same slide using M-FISH probes (Metasystems Gmbh, Althusseim, Germany), according to the manufacturer’s recommendations. Images of hybridized metaphases were captured using a charge coupled device camera (Zeiss, Thornwood, NY, USA) coupled to a Zeiss Axioplan microscope and processed using ISIS software (Metasystems Gmbh, Althusseim, Germany) [39].

#### 4.2.7. Telomerase Activity and Telomere Maintenance Mechanisms in Clonogenic Cells

The PCR-based telomere repeat amplification protocol (TRAP) assay of telomerase enzyme activity using the TeloTAGGG™ Telomerase PCR ELISA PLUS (Roche-12013789001) was carried described according to manufacturer’s instructions. Equal cell numbers (10^6^ cells) were used for each experiment and relative telomerase activity was calculated based on the value of two differences in CT value. Immunofluorescence for hTERT (Sigma, Lezennes, France) was performed to confirm the TRAP results. Co-immunofluorescence staining of hTERT and PML (InterBiotech Interchim, Montliçon, France) was performed to detect the mosaicism concerning telomere maintenance mechanisms in HL cells.

#### 4.2.8. Statistical Analysis

Linear regression was performed to fit the data (slope and intercept) using the least squares method. The significance test for linear regression (*p*-value and squared-R) were performed using the R command including the Fisher Test for regression. Kaplan-Meier method was also performed.

## 5. Conclusions

We have established a novel HL animal model that requires only 10^3^ cells for injection. Moreover, it is characterized by a high survival/toxicity ratio and high reproducibility. The originality of this model is based on the use of cell proliferation in semi-solid medium, which are expanded in vitro, before transplantation into mice. We demonstrated the presence of a high frequency of small cells lacking HL markers characterized by higher telomerase expression and telomere dysfunction in in vivo tumors. Molecular characterization of these negative cells may establish how transformation evolves from CD15/CD30-negative cells to Hodgkin and HRS cells. It will be informative to test this same approach, using cells derived and amplified from experimental in vivo tumors, as well as those derived from human circulating lymphocytes or tumor tissues from relapsed/refractory HL patients.

Telomere dysfunction appears to play a major role in the genomic instability observed in HL cells, and possibly, through “intrinsic” aberrations, in the oncogenesis of HL.

## Figures and Tables

**Table 1:** Patient’s characteristics.

Characteristics	N° of Patients	%
	(*N* = 50)	
**Male**	31	62
**Age**	35 y	
<45	40	80
>45	10	20
**Stage**		
Stage I	10	20
Stage II	35	70
Stage II	5	10
**Treatment**		
Chemotherapy only	0	0
Combined modality	50	100
**Histological sub-type**		
Nodular Sclerosis	36	94.7
Mixed cellularity	6	2.6
Classic lymphocyte rich	4	2.6

## Supplementary Materials

The following are available online at http://www.mdpi.com/2072-6694/10/11/414/s1. Figure S1. Characterization of CD30/CD15 immuno-phenotypes of HL cell lines (A) FACS analysis demonstrates considerable variability in CD30 and CD15 expression between different HL cell lines. (B) Immunofluorescence microscopy of CD30 stained HL cell lines validates the results obtained by flow cytometry and confirms the presence of CD30− cells. Table S1. In vitro colony formation of HL cell lines in semi-solid medium (methylcellulose) after the first and second HL cloning experiment. Figure S2: In vitro cell proliferation of clones derived from the L428 cell line in methylcellulose showing higher in vitro proliferation of selected clones than the parental cell line. Figure S3. Proliferation of CD30−/CD15− cells in vitro after fluorescence activated cell sorting of the L540 cell line. (A) Representative dot plots showing the gating strategy using L540 cells without any staining to fix the gating (B) L540 cells were stained for CD15 and CD30 and sorted: CD30−/CD15− cells (yellow inset), CD30+/CD15− cells (red inset), and CD30+/CD15+ cells (green inset). (C) Validation of the cell sorting using the CD30+/CD15− population analyzed by FACScan flow cytometer. (D) Analysis of L540 cell line after CD30/CD15 staining using FACScan flow cytometer showing different sub populations regarding CD30 and CD15 expression. FSC of different subpopulations were similar to that observed in L428-c subline. Small cells were CD30−/CD15−. (E) CD30−/CD15− subpopulation after three weeks in culture (F) CD30−/CD15− subpopulation after four weeks in culture, showing a similar profile as that of the parental cells. Table S2. In vivo infiltration of unsorted HL cell lines in NSG mice compared to that obtained using the L428-c subline. Figure S4. Flow cytometry and immunofluorescence analysis of cells recovered from tumors of two mice injected with 10^3^ L428-c cells. The recovered cells were analyzed eight weeks after injection. (A) Flow cytometry analysis revealed a higher frequency of CD30-cells. (B) Immunofluorescence analysis of CD30 (red signal) confirmed the presence of small CD30-cells. Figure S5. Mosaic of chromosomal aberrations detected in L428-c after two weeks and five weeks of in vitro expansion. (A) Small cells detected at two weeks showed the presence of der(5)t(X;5;9;5) associated with the presence of r(2), dic(3;15); dic(5;13); dic(11;19), and dic(X;21). (B) A similar karyotype was found in the L428-c subline after five weeks of in vitro expansion together with the other aberrations identified in HL cells that were derived from the livers of mice. Figure S6. hTERT (green) and PML (red) expression in HL cells derived from mouse livers at (A) five weeks, (B) 16 weeks, and (C) 32 weeks of in vivo expansion, showing higher hTERT expression in small cells and the presence of PML bodies in HRS cells. Figure S7. EDO-S101-induced apoptosis in HL cell lines following 48 h of in vitro exposure. AnnexinV-FITC and Hoechst double staining were performed. Only viable cells were analyzed and all necrotic cells were excluded. For each condition and each cell line, 10,000 events were analyzed. The L428-c subline showed higher sensitivity to EDO-S101than the parental L428 cells. Figure S8. EDO-S101 exposure combined with ionizing radiation. After 48 h of EDO-S101 exposure, HL cell lines were irradiated at 6 Gy. Following 24 h and using cell cycle arrest analysis, we observed altered cell-cycle profiles, suggesting sensitivity of the HL cell lines to irradiation and/or EDO-S101 exposure. The L428-c subline showed increased radiation sensitivity after EDO-S101 exposure, as well as after irradiation. G2 arrest was observed in all HL cell lines. Figure S9. Common steps in the flow cytometry analysis, four samples were analyzed for each condition. (A) cells without staining in order to fix the gating and autofluorescence standard cells are subsequent identified in the two channels (FITC for CD30 and red for CD15). (B) Cells stained with only CD30 and the fluorescence intensity of FITC spectrum was measured as well as the intensity of red channel (control). (C) Cells stained with only CD15 and fluorescence intensity of red signal was measured as well as the intensity of FITC signal. (D) Cells were stained with CD30 and CD15 and the intensity of the two channels are presented. FSC-SSC plot, in which the gates for controls and staining cells are delimited. The analysis of L428 cell line is presented.
