# Supplementary material for: Establishment and Characterization of a Reliable Xenograft Model of Hodgkin Lymphoma Suitable for the Study of Tumor Origin and the Design of New Therapies
Source: Cancers (Basel). 2018 Oct 31;10(11):414. doi: 10.3390/cancers10110414 (PMC6265845; doi:10.3390/cancers10110414)
Supplement: Supplementary file 1 [file cancers-10-00414-s001.pdf]

# Supplementary Materials: Establishment and Characterization of a Reliable Xenograft Model of Hodgkin Lymphoma Suitable for the Study of Tumor Origin and the Design of New Therapies

**Radhia M'kacher, Monika Frenzel, Mustafa Al Jawhari, Steffen Junker, Corina Cuceu, Luc Morat, Anne-Laure Bauchet, Lev Stimmer, Aude Lenain, Nathalie Dechamps, William M. Hempel, Geraldine Pottier, Leonhard Heidingsfelder, Eric Laplagne, Claire Borie, Noufissa Oudrhiri, Dima Jouni, Annelise Bennaceur-Griscelli, Bruno Colicchio, Alain Dieterlen, Theodore Girinsky, Raphael Boisgard, Jean Bourhis, Jacques Bosq, Thomas Mehrling, Eric Jeandidier, Patrice Carde**

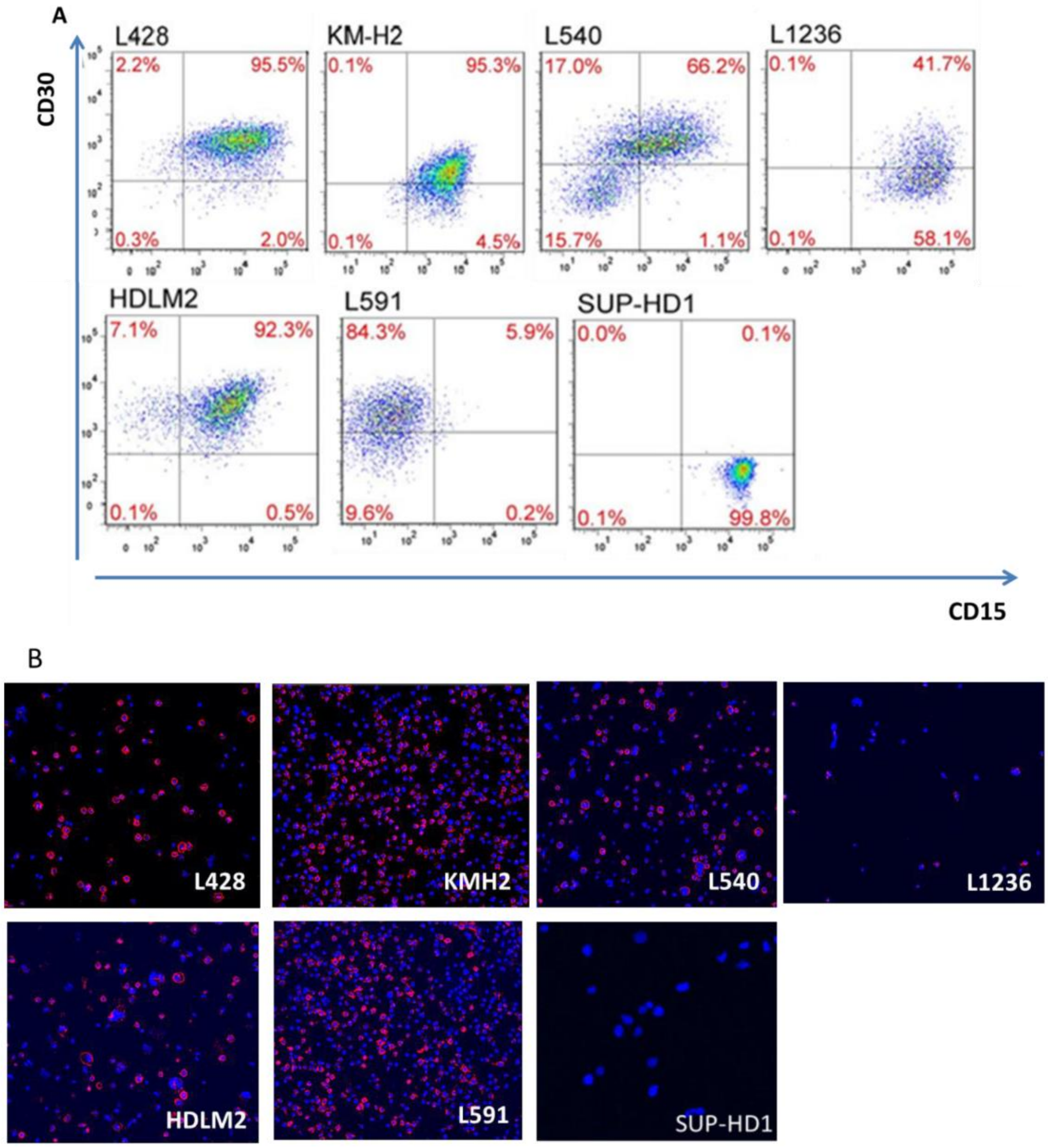


**Figure S1.** Characterization of CD30/CD15 immuno-phenotypes of HL cell lines (**A**) FACS analysis demonstrates considerable variability in CD30 and CD15 expression between different HL cell lines. (**B**) Immunofluorescence microscopy of CD30-stained HL cell lines validates the results obtained by flow cytometry and confirms the presence of CD30− cells.

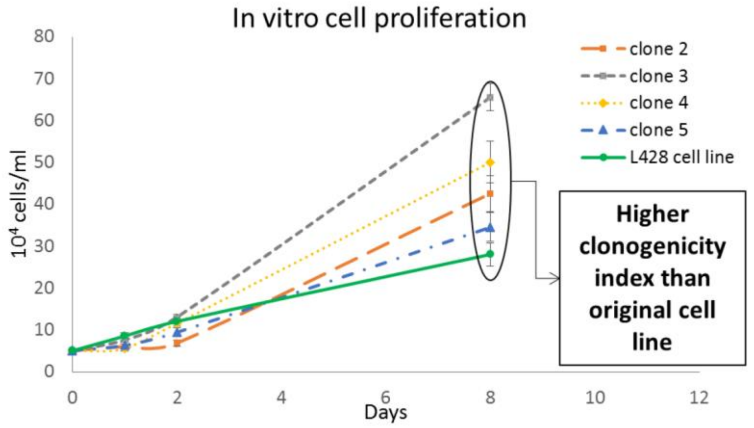


**Figure S2.** In vitro cell proliferation of clones derived from the L428 cell line in methylcellulose. Higher in vitro proliferation of selected clones compared to parental cell line.

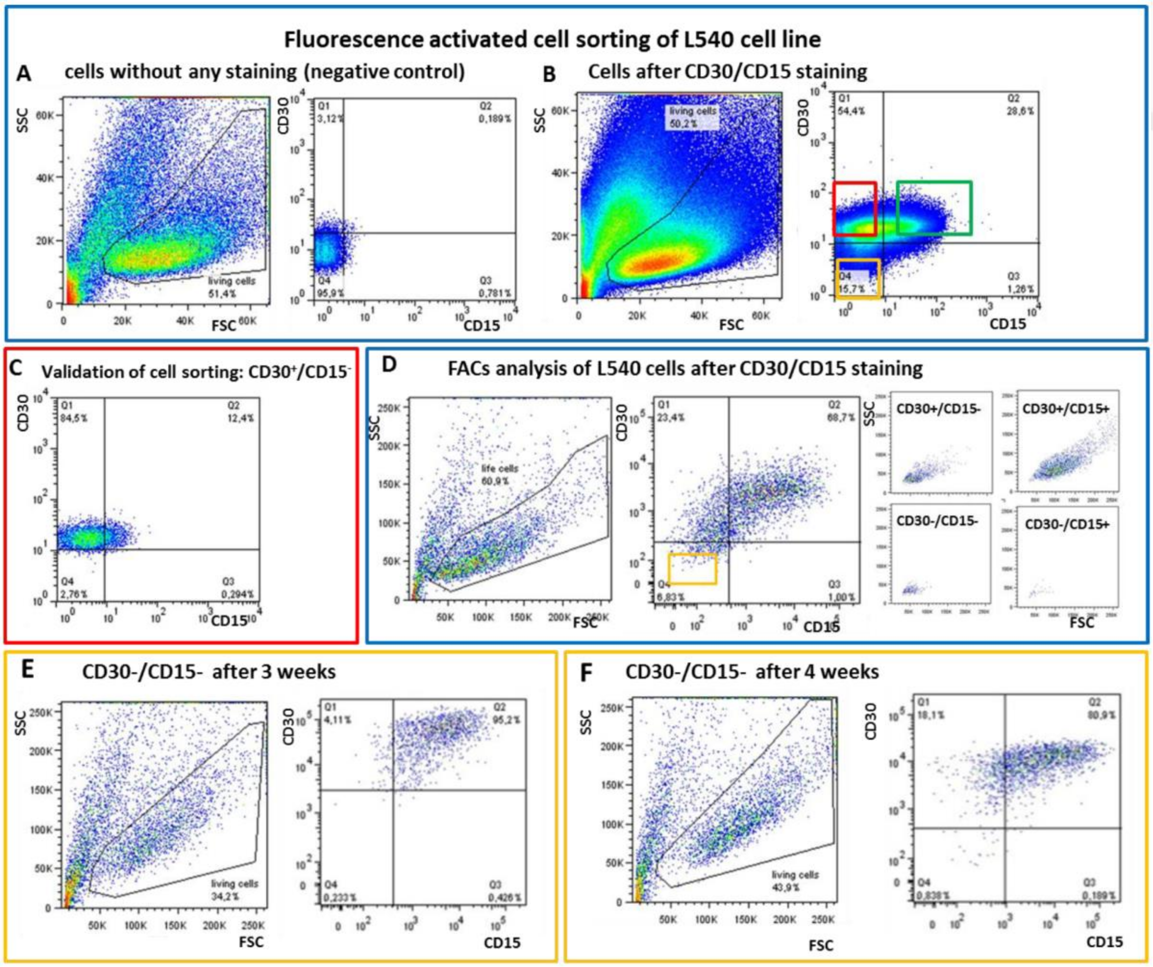


**Figure S3.** Proliferation of CD30−/CD15− cells *in vitro* after fluorescence activated cell sorting of L540 cell line. (**A**) Representative dot plots showing gating strategy using L540 cells without any staining to fix the gating (**B**) L540 cells were stained for CD15 and CD30, and CD30−/CD15− cells (yellow inset) were collected as well as CD30+/CD15− cells (red inset) and CD30+/CD15+ cells (green inset). (**C**) The validation of cell sorting using CD30+/CD15− population analyzed by FACScan flow cytometer. (**D**) Analysis of L540 cell line after CD30/CD15 staining using FACScan flow cytometer showing different sub populations regarding CD30 and CD15 expression. FSC of different subpopulations were similar to that observed in L428-c subline. Small cells were CD30-/CD15−. (**E**) CD30−/CD15− subpopulation

after three weeks in culture (**F**) CD30−/CD15− subpopulation after four weeks in culture showing a similar profile as that of the parental cells.

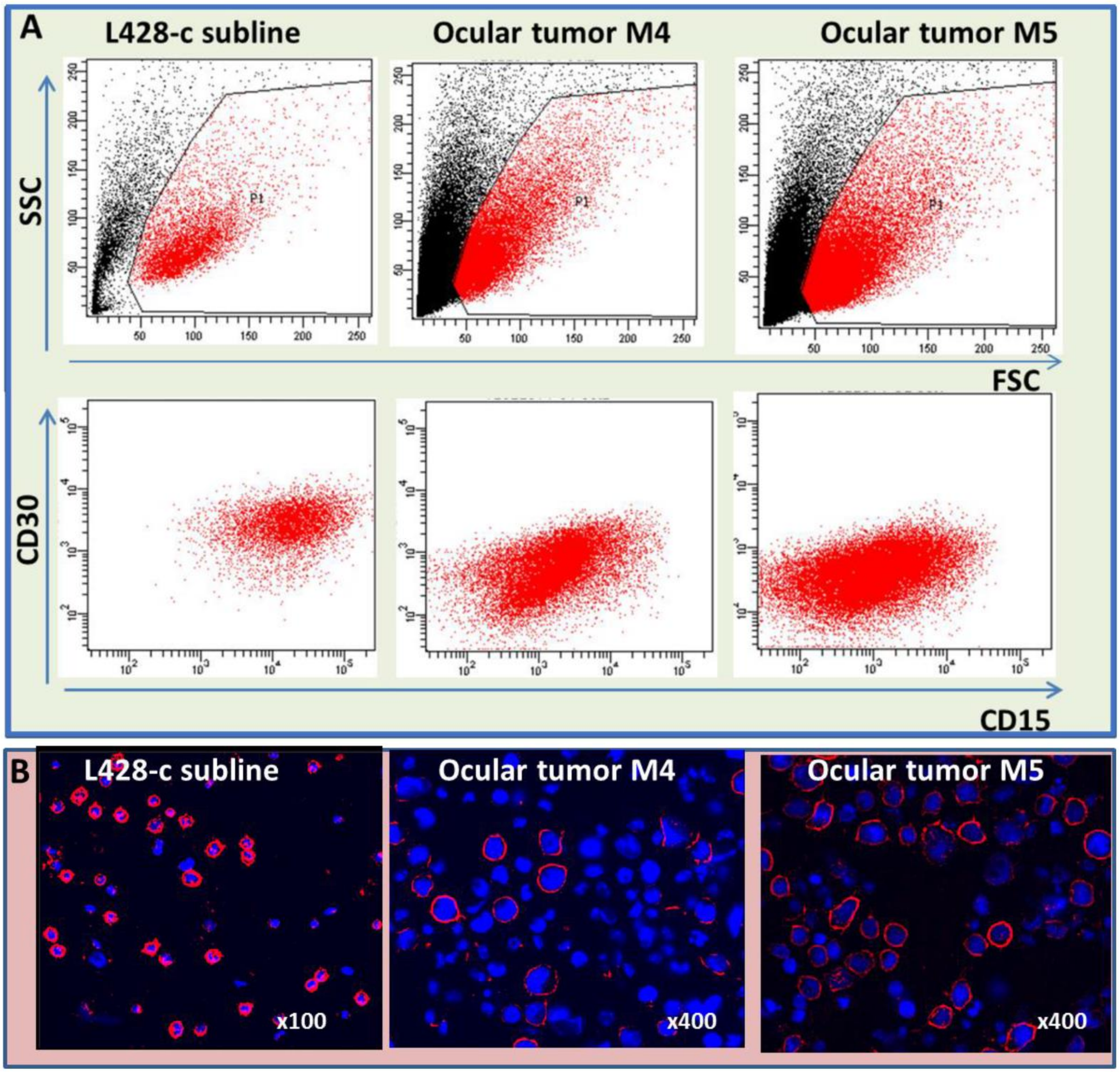


**Figure S4.** Flow cytometry and immunofluorescence analysis of cells recovered from tumors of two mice injected with 103 L428-c cells. The recovered cells were analyzed eight weeks after injection. (A) Flow cytometry analysis revealed a higher frequency of CD30-cells. (B) Immunofluorescence analysis of CD30 (red signal) confirmed the presence of small CD30-cells.

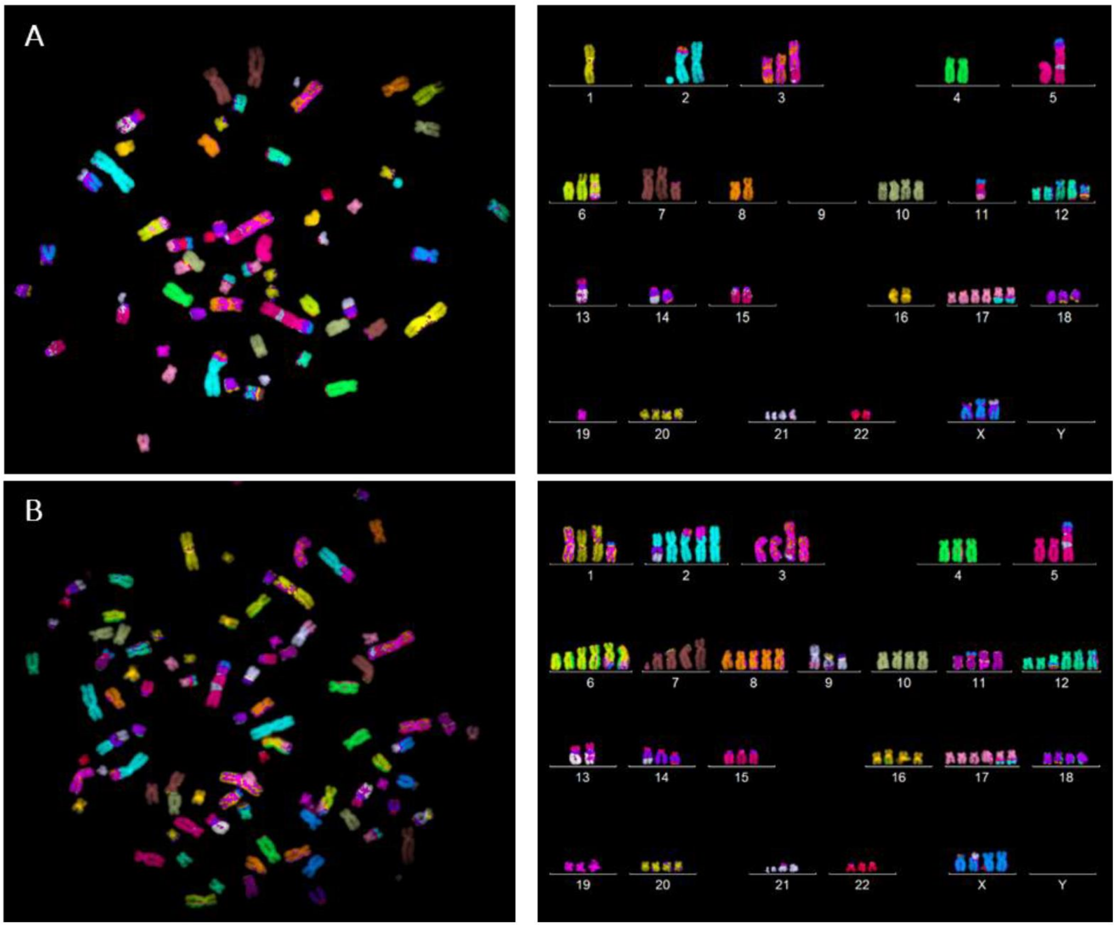


**Figure S5.** Mosaic of chromosomal aberrations detected in L428-c after two weeks and five weeks of in vitro expansion. (**A**) Small cells detected at two weeks showed the presence of der(5)t(X;5;9;5) associated with the presence of r(2), dic(3;15); dic(5;13); dic(11;19), and dic(X;21). (**B**) A similar karyotype was found in the L428-c subline after five weeks of in vitro expansion together with the other aberrations identified in HL cells that were derived from the livers of mice.

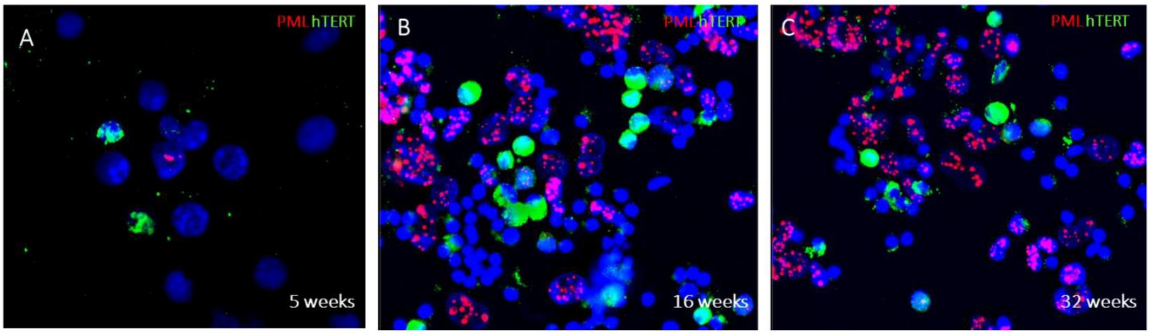


**Figure S6.** hTERT (green) and PML (red) expression in HL cells derived from mouse livers at (**A**) five weeks, (**B**) 16 weeks, and (**C**) 32 weeks of in vivo expansion, showing higher hTERT expression in small cells and the presence of PML bodies in HRS cells.

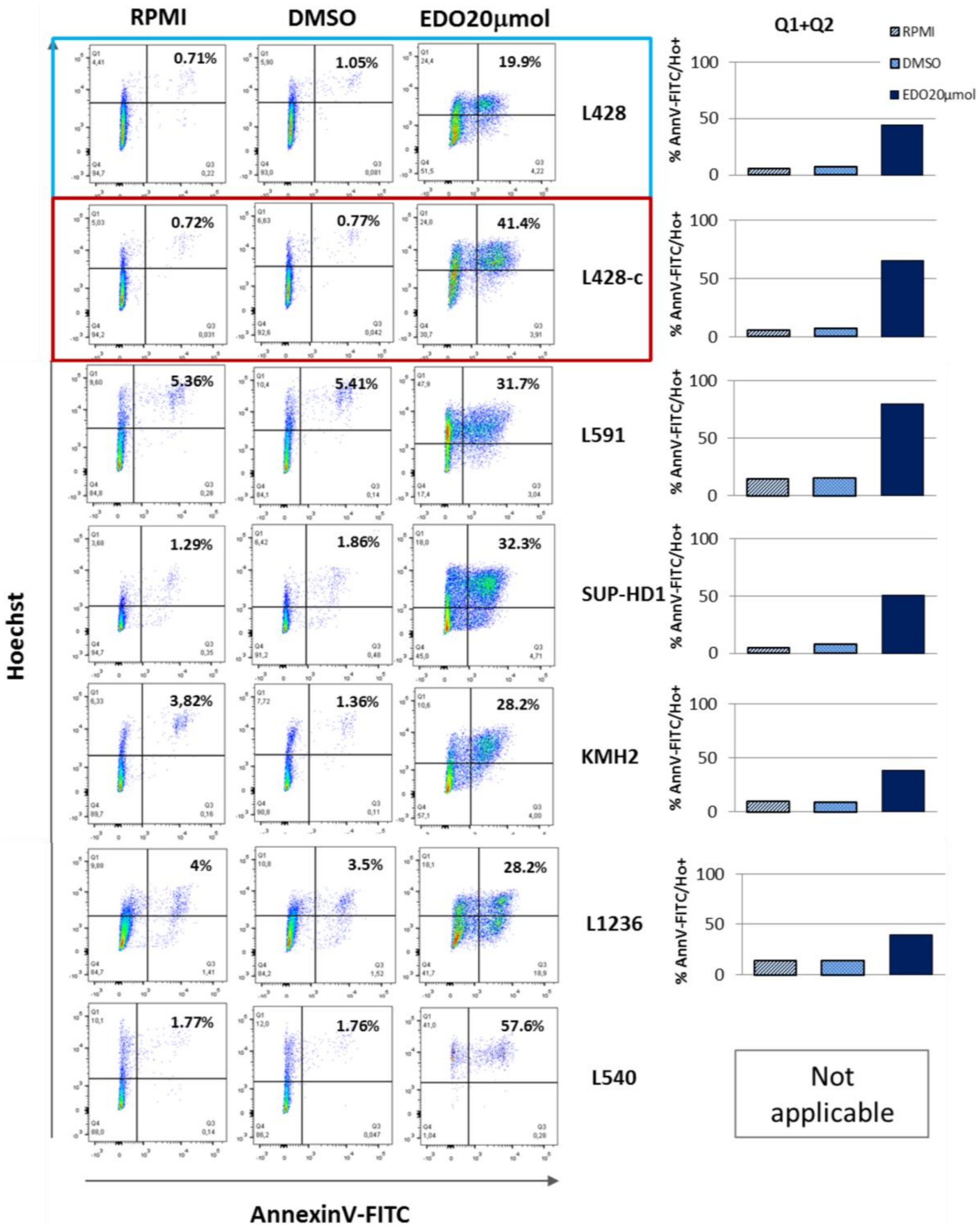


**Figure S7.** EDO-S101-induced apoptosis in HL cell lines following 48 h of in vitro exposure. AnnexinV-FITC and Hoechst double staining were performed. Only viable cells were analyzed and all necrotic cells were excluded. For each condition and each cell line, 10,000 events were analyzed. The L428-c subline showed higher sensitivity to EDO-S101 than the parental L428 cells.

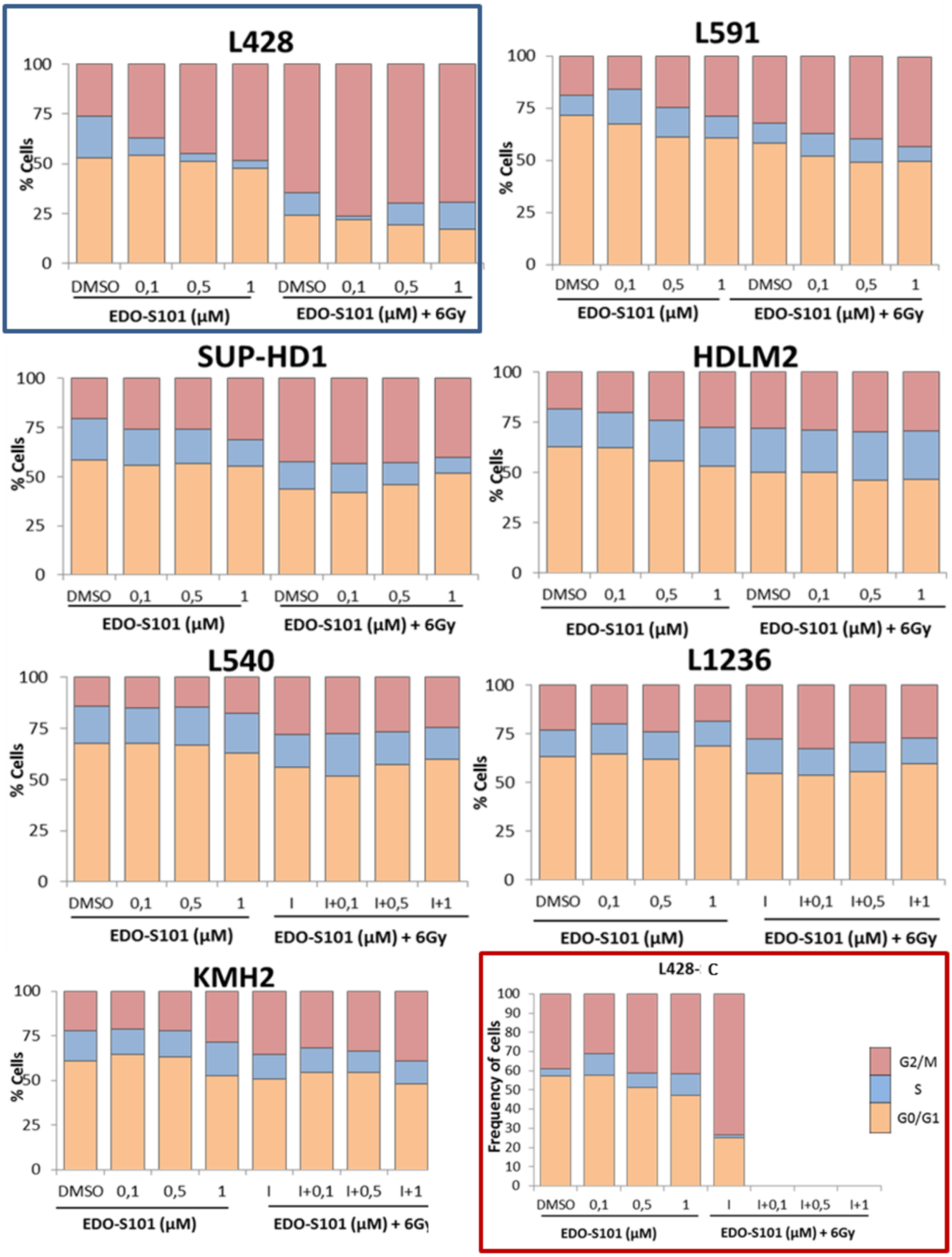


**Figure S8.** EDO-S101 exposure combined with ionizing radiation. After 48 h of EDO-S101 exposure, HL cell lines were irradiated at 6 Gy. Following 24 h and using cell cycle arrest analysis, we observed altered cell-cycle profiles, suggesting sensitivity of the HL cell lines to irradiation and/or EDO-S101 exposure. The L428-c subline showed increased radiation sensitivity after EDO-S101 exposure, as well as after irradiation. G2 arrest was observed in all HL cell lines.

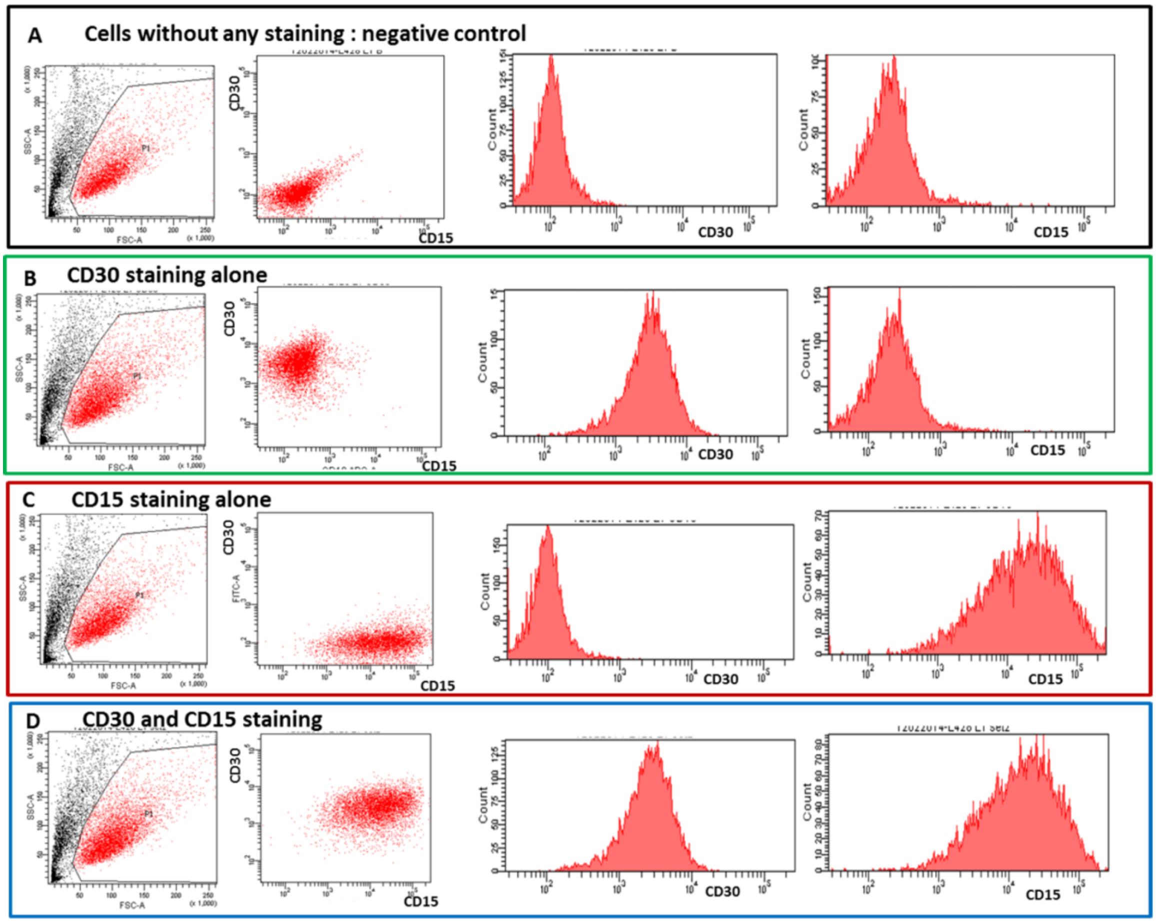


**Figure S9.** Common steps in the flow cytometry analysis, four samples were analyzed for each condition. (**A**) cells without staining in order to fix the gating and autofluorescence standard cells are subsequent identified in the two channels (FITC for CD30 and red for CD15). (**B**) Cells stained with only CD30 and the fluoresence intensity of FITC spectrum was measured as well as the intensity of red channel (control). (**C**) Cells stained with only CD15 and fluoresence intensity of red signal was measured as well as the intensity of FITC signal. (**D**) Cells were stained with CD30 and CD15 and the intensity of the two channels are presented. FSC-SSC plot, in which the gates for controls and satining cells are delimited. The analysis of L428 cell line is presented.

**Table S1.** In vitro colony formation of HL cell lines in semi-solid medium (methylcellulose) after the first and second HL cloning experiment.

| HL cell lines | Colony formation, | First plating No. of colonies/$10^5$ cells, mean±σ | Second plating No. of colonies /$10^5$ cell, mean ±σ |
|---|---|---|---|
| **L428** | +++ | 188,5±18 | 923±13 |
| **KMH2** | +++ | 169±20 | 680±14 |
| **L540** | +++ | 103±15 | 420±19 |
| **L1236** | + | 6±2 | - |
| **L591** | - | - | - |
| **SUP-HD1** | + | 10±3 | - |
| **HDLM2** | + | 5±2 | - |

**Table S2.** In vivo infiltration of unsorted HL cell lines in NSG mice compared to that obtained using the L428-c subline.

| Cell line | No of cells inoculated/ mouse($10^6$) | Time of sacrifice (days after inoculation) | N° of mice with tumor/no of mice inoculated | Infiltration of HL cells into: | | | |
|---|---|---|---|---|---|---|---|
| | | | | Liver | Spleen | Bone Marrow | Eye* |
| **L428-c** | $10^6$ | 35 | 12/12 | +++ | ++ | ++ | +++ |
| **L428** | $10^6$ | 35 | 7/9 | +++ | ++ | ++ | +++ |
| **KMH2-c** | $10^6$ | 35 | 6/9 | +++ | ++ | ++ | +++ |
| **L540-c** | $10^6$ | 90 | 6/9 | +++ | ++ | ++ | ++ |
| **L1236** | $10^6$ | 120 | 1/4 | - | - | - | ++ |
| **L591** | $10^6$ | 60 | 0/4 | - | - | - | - |

* Site of injection.

© 2018 by the authors. Licensee MDPI, Basel, Switzerland. This article is an open access article distributed under the terms and conditions of the Creative Commons Attribution (CC BY) license (http://creativecommons.org/licenses/by/4.0/).
